# The interplay between serine proteases and caspase-1 regulates the autophagy-mediated secretion of Interleukin-1 beta in human neutrophils

**DOI:** 10.3389/fimmu.2022.832306

**Published:** 2022-08-25

**Authors:** Irene A. Keitelman, Carolina M. Shiromizu, Nadia R. Zgajnar, Silvia Danielián, Carolina C. Jancic, Marcelo A. Martí, Federico Fuentes, Judith Yancoski, Douglas Vera Aguilar, David A. Rosso, Verónica Goris, Guadalupe Buda, María Martha Katsicas, Mario D. Galigniana, Jeremías G. Galletti, Florencia Sabbione, Analia S. Trevani

**Affiliations:** ^1^ Laboratorio de Inmunidad Innata, Instituto de Medicina Experimental (IMEX) - CONICET, Academia Nacional de Medicina, Buenos Aires, Argentina; ^2^ Laboratorio de receptores nucleares, Instituto de Biología y Medicina Experimental (IBYME)-CONICET, Buenos Aires, Argentina; ^3^ Laboratorio de Biología Molecular Inmunología, Hospital de Pediatría “Juan P. Garrahan”, Buenos Aires, Argentina; ^4^ Departamento de Microbiología, Parasitología e Inmunología, Facultad de Medicina, Universidad de Buenos Aires, Buenos Aires, Argentina; ^5^ Departamento de Química Biológica, Facultad de Ciencias Exactas y Naturales (FCEyN), Universidad de Buenos Aires (UBA), Buenos Aires, Argentina; ^6^ Instituto de Química Biológica de la Facultad de Ciencias Exactas y Naturales (IQUIBICEN) – CONICET, Facultad de Ciencias Exactas y Naturales (FCEyN), Universidad de Buenos Aires (UBA), Buenos Aires, Argentina; ^7^ Laboratorio de Microscopía, Instituto de Medicina Experimental (IMEX) - CONICET, Academia Nacional de Medicina, Buenos Aires, Argentina; ^8^ Unidad de Genómica. Laboratorio de Biología Molecular de Inmunología, Hospital de Pediatría “Juan P. Garrahan”, Buenos Aires, Argentina; ^9^ Servicio de Inmunología y Reumatología, Hospital de Pediatría “Juan P. Garrahan”, Buenos Aires, Argentina

**Keywords:** autophagosome, granulocytes, inflammasome, neutral proteases, unconventional protein secretion

## Abstract

Neutrophils play major roles against bacteria and fungi infections not only due to their microbicide properties but also because they release mediators like Interleukin-1 beta (IL-1β) that contribute to orchestrate the inflammatory response. This cytokine is a leaderless protein synthesized in the cytoplasm as a precursor (pro-IL-1β) that is proteolytically processed to its active isoform and released from human neutrophils by secretory autophagy. In most myeloid cells, pro-IL-1β is processed by caspase-1 upon inflammasome activation. Here we employed neutrophils from both healthy donors and patients with a gain-of-function (GOF) *NLRP3-*mutation to dissect IL-1β processing in these cells. We found that although caspase-1 is required for IL-1β secretion, it undergoes rapid inactivation, and instead, neutrophil serine proteases play a key role in pro-IL-1β processing. Our findings bring to light distinctive features of the regulation of caspase-1 activity in human neutrophils and reveal new molecular mechanisms that control human neutrophil IL-1β secretion.

## Introduction

Neutrophils are key actors of the innate immune system that play a major role as the first line of defense against infections (1). These granulocytes, the most abundant leukocytes in human circulation, are massively recruited to infection foci where they deploy a battery of antimicrobial weapons to fight against the potentially harmful invaders. The crucial role of neutrophils in the immune response is evidenced by the severe, and often fatal, course of infections in patients with congenital neutrophil deficiencies.

Neutrophils are terminally differentiated cells with a short lifespan that kill microbes by phagocytosis and extracellular traps formation. However, their role in the immune system goes far beyond these functions. These cells also contribute to coordinate the immune response by means of the release of preformed, and *de novo* synthesized proinflammatory mediators (2). Among them, is the highly inflammatory cytokine Interleukin-1 beta (IL-1β) which plays major roles in host defense and immune pathology (3).

Neutrophils are characterized by the presence of heterogeneous granules containing a variety of enzymes and anti-microbial peptides that exert key functions as part of the non-oxidative arm of their microbicidal actions. Among these granules, the azurophilic ones contain the potent neutral serine proteases elastase (NE), proteinase 3 (PR3), cathepsin G (CG), and neutrophil serine protease 4 (NSP4) (4).

Neutrophil serine proteases (NSPs) are synthesized during the promyelocytic stage of granulopoiesis in the bone marrow, and under homeostatic conditions, they are stored within the azurophilic granules of circulating neutrophils (5). Upon microbe internalization, azurophilic granules fuse with phagosome membranes in a process that licenses these enzymes to degrade the internalized cargo. Also, granule binding to plasma membrane allows serine proteases to have extracellular microbicidal action as well as tissue-degrading effects. Cumulative evidence indicates that NSPs also exert other actions outside granules and phagosomes either intracellularly or extracellularly (6). In fact, in aging neutrophils, PR3 is released to the cytosol *via* lysosomal membrane permeabilization, an event that leads to pro-caspase-3 cleavage and apoptosis. The cytosolic PR3 activity is counteracted by the suicide protease inhibitor SERPINB1. The final balance between PR3 and SERPINB1 controls neutrophil spontaneous death (7). Furthermore, PR3 is also expressed bound to CD177 on the plasma membrane of freshly isolated neutrophils. There is also evidence that not only PR3 but also NE and CG can associate with the plasma membrane under inflammatory conditions (6). On the other hand, upon stimulation with agonists that induce neutrophil extracellular traps formation, NE escapes from azurophilic granules through a reactive oxygen species (ROS)-dependent process (8). In the cytosol, NE binds and degrades F-actin to arrest actin dynamics, and subsequently it translocates to the nucleus, where it partially degrades specific histones promoting chromatin decondensation (9). Recent studies also determined that N-Gasdermin D (N-GSDMD), the pore-forming fragment of Gasdermin D generated by active caspase-1, in neutrophils traffics to the membrane of azurophilic granules, thus mediating the leakage of neutrophil elastase into the cytosol. This event, in turn, facilitates a secondary cascade of serine protease-dependent GSDMD processing (10).

Interleukin-1β is a pro-inflammatory cytokine that lacks a signal peptide. It is synthesized as an inactive precursor (pro-IL-1β) in the cytosol, where it is processed by enzymatic cleavage. In myeloid cells, the precursor processing usually relies on the action of the enzyme caspase-1, which in turn is activated upon the formation of the intracellular macromolecular complexes called inflammasomes (11, 12). The canonical inflammasomes comprise an inflammasome sensor protein, such as the nucleotide-binding domain and leucine-rich-repeat containing protein 3 (NLRP3), which binds to caspase-1 usually *via* the adapter ASC (apoptosis-associated speck-like protein containing the caspase activation and recruitment domain [CARD]). NLRP3 is activated by pathogen- and danger-associated molecular patterns representative of infection, cellular damage, or cell stress, which promotes its oligomerization (13). This, in turn, leads to ASC recruitment and polymerization, originating an ASC-speck structure, to which full-length caspase-1 binds. Full-length caspase-1 dimerization in the inflammasome structure triggers its protease activity, which allows its self-cleavage to generate a fully active p33/p10 species, which remains bound to the inflammasome and is responsible for pro-IL-1β processing, i.e., the generation of the mature 17-kDa IL-1β isoform (14). Then, caspase-1 self-cleaves again generating a p20/p10 species. This cleavage releases caspase-1 from the inflammasome and terminates its protease activity and cytokine processing (3, 14).

Interestingly, other studies showed that NSPs can also process pro-IL-1β into a bioactive fragment and attributed a role to these enzymes in the extracellular processing of pro-IL-1β released at inflammatory environments (15–17).

Previously we determined that highly purified human neutrophils produce and secrete IL-1β (18, 19). These studies showed that human neutrophil IL-1β secretion is restrained by both caspase-1 and NSPs inhibition, and that cytokine secretion, but not pro-IL-1β processing, is dependent on NADPH oxidase-derived ROS (18). As pro-IL-1β is a leaderless protein synthesized in the cytosol, once processed, IL-1β is usually released by unconventional secretory mechanisms. Our previous studies also determined that human neutrophils release IL-1β by an autophagy-mediated secretory mechanism (19). In fact, pharmacological inhibition of autophagy as well as ATG5 knockdown, reduced IL-1β secretion, while autophagy stimulation by starvation increased it. Additional studies performed by using ATG7-deficient mouse cells confirmed these findings (10).

Considering that both caspase-1 and NSPs are required for the release of active IL-1β, we conducted this study to address the role of these enzymes in the mechanisms involved in pro-IL-1β processing in human neutrophils and those leading to IL-1β secretion. By studying the responses of human neutrophils from healthy donors and from patients with a GOF *NLRP3*-mutation, we found that even though caspase-1 is activated after inflammasome activation and is required for IL-1β secretion, it mostly undergoes rapid inactivation and NSPs accomplish a major role in pro-IL-1β processing.

## Materials and methods

The experimental protocols performed have been approved by the Biosafety and Research Review boards of the “Instituto de Medicina Experimental (IMEX)-CONICET-Academia Nacional de Medicina” and the Ethical Committee of the “Institutos de la Academia Nacional de Medicina”.

### Reagents and materials

RPMI 1640 culture medium, Earle’s Balanced Salt Solution (EBSS), LysoTracker™ Red DND‐99, ammonium persulfate, Pierce LDH Cytotoxicity Assay Kit, dextran 500, and TMB substrate were purchased from Thermo Fisher Scientific Life Technologies (MA, USA). Fetal bovine serum (FBS) and bovine serum albumin were purchased from Internegocios (Buenos Aires, Argentina). Ficoll-Paque was purchased from GE Healthcare (Munich, Germany). BD OptEIA™ Human IL-1β ELISA Set II and Human IL-8/CXCL8 ELISA Set were purchased from BD Biosciences (Franklin Lakes, NJ, USA). Quantikine Human Pro-IL-1β/IL-1F2 Immunoassay and Human IL-1β/IL-1F2 Propeptide Antibody (MAB6964) were purchased from R&D (Minneapolis, MN, USA). Secondary antibodies were purchased from Jackson Immunoresearch Laboratories (West Grove, PA, USA): Alexa Fluor^®^ 647 AffiniPure F(ab’)_2_ Fragment Goat Anti-Rabbit IgG (H + L), cat. #111-606-144; Alexa Fluor^®^ 488 AffiniPure F(ab’)_2_ Fragment Goat Anti-Rabbit IgG (H + L) cat. #111-546-144; DyLight 549 conjugated AffiniPure F(ab’)_2_ Fragment Goat Anti-mouse IgG (H + L), cat. #115-506-062; Cy™3 AffiniPure F(ab’)2 Fragment Goat Anti-Mouse IgG (H+L), cat. #115-166-146. TO-PRO-3 was obtained from Life Technologies (Carlsbad, CA, USA). Phycoerythrin-conjugated anti-CD14 antibody was purchased from eBioscience (San Diego, CA, USA). Aqua Poly/Mount Coverslipping Medium was purchased from Polysciences (Warrington, PA, USA). Rabbit polyclonal antibody anti-microtubule-associated protein 1 light chain 3 beta (LC3B) cat. #sc28266; rabbit polyclonal antibody anti-IL-1 cat. #sc7884, anti-caspase-1 p10(C-20) cat. #515 antibody and cat. #sc-56036 were from Santa Cruz Biotechnology (Dallas, TX, USA); rabbit polyclonal anti-human IL-1 beta cat. NB600-633 was from Novus Biologicals (Co, USA); rabbit polyclonal anti-human-myeloperoxidase cat.#A0398 was from Dako (Glostrup, Denmark); rabbit polyclonal anti-sequestosome 1(SQSTM1)/p62 cat.# F48010 was from NSJ Bioreagents (San Diego, CA, USA); and purified mouse IgG1, κ isotype control cat. #555746 and rabbit polyclonal IgG was purchased from Jackson Immunoresearch. Complete™, EDTA-free Protease Inhibitor Cocktail, cat. #11873580001 was from Roche (Basel, Switzerland). Hyperfilm ECL Amersham and Hybond PVDF membrane were from Amersham Cytiva (Marlborough, MA, US). AEBSF (4-(2-aminoethyl)-benzenesulfonyl fluoride, monohydrochloride), Ac-YVAD-CMK (N-acetyl-L-tyrosyl-L-valyl-N-[(1S)-1-(carboxymethyl)-3-chloro-2-oxo-propyl]-L-alaninamide), Z-VAD-FMK, Bafilomycin A1, and 3-methyladenine (3-MA) and VX-765 were purchased from Cayman Chemical (Michigan, USA), Annexin V-FITC cat. # 31490013 was purchased from Immunotools (Gladiolenweg, Germany). FAM‐FLICA^®^ Caspase‐1 Assay Kit and FAM‐FLISP FLCK Serine protease kit were purchased from Immunochemistry Technologies (Bloomington, MN, USA). All other chemicals employed were purchased from Sigma Aldrich (St. Louis, MO, USA).

### Human neutrophil isolation

Cells were isolated from ACD-anticoagulated human blood from healthy adult donors or from patients with the gain-of-function (GOF) *NLRP3* mutation c.1322C>T (p.Ala441Val) validated as Pathogenic on the Infevers Database, classification proposed by the International Study Group for Systemic Autoinflammatory Diseases (20) and aged-matched control donors. Neutrophils were isolated by centrifugation on Ficoll-Paque, dextran sedimentation, and hypotonic lysis. Cells were suspended at 6 × 10^6^/mL in RPMI 1640 supplemented with penicillin (100 U/mL), streptomycin (100 μg/mL), and 10% FBS. After isolation, neutrophil preparations were stained with an anti-CD14-PE antibody and analyzed with a Partec Cyflow cytometer to guarantee that monocyte contamination was less than 0.5% as previously described (19). Cells were used immediately after isolation.

### Neutrophil stimulation

Neutrophils (5x10^6^/ml) were treated for 2 h with or without 150 ng/mL LPS from *Escherichia coli* O111:B4. Then, they were stimulated or not with 2.5 mM ATP and cultured for the specified times. Where indicated, before LPS stimulation, cells were pretreated for 30 min with AEBSF (1 mM or 0.35 mM) and/or Ac-YVAD-CMK (50 µM), VX-765 (50 µM) or Z-VAD-FMK (50 µM). After culture, cell supernatants, and where indicated, also the cell pellets, were collected and pro-IL-1β, IL-1β and IL-8 concentrations were quantitated by ELISA. In cell pellets, viability was determined by annexin V-FITC/propidium iodide (PI) staining and flow cytometry analysis. Alternatively, at the specified time, cell pellets were fixed with 4% paraformaldehyde (PFA) and processed for either confocal laser scanning microscopy (CLSM) or flow cytometry. In some experiments, inhibitors were added at different time points after ATP treatment and at 5 h post-LPS stimulation, supernatants and pellets were collected and IL-1β was determined by ELISA.

### Intracellular immunostainings and CLSM acquisition

After fixation with PFA 4% for 30 min, cells were blocked with PBS-glycine (0.1 M) for 15 min, permeabilized with chilled acetone (−20°C) for 7 min, rehydrated with PBS and blocked with PBS supplemented with 5% goat serum overnight at 4°C. Then, neutrophils were incubated with the primary antibodies in blocking buffer for 1 h at room temperature, washed, and then incubated with the corresponding secondary antibodies for 1 h at room temperature. In some experiments TO-PRO-3 was added for nuclei staining. Then cells were washed, cyto-spinned, mounted with Aqua-Poly/mount mounting medium, and stored at 4°C until microscopy examination. Image acquisition was performed by using a FluoView FV1000 confocal microscope (Olympus, Tokyo, Japan) equipped with a Plapon 60X/1.42 objective. Images were analyzed with ImageJ software (NIH) and fluorescence was quantitated. Some CLSM experiments were performed by seeding neutrophils on poly-L-lysine coated Lab-Tek chambers (Nalge Nunc International, New York, NY, USA).

### Phalloidin staining

Cells were fixed with PFA 4% for 20 min, then stained with Phalloidin-TRITC (50 µg/mL) for 40 min and washed thoroughly with PBS. Next, cells were subjected to flow cytometry or CLSM image acquisition.

### Flow cytometry analysis

For certain experiments, after immunostaining, cell fluorescence was determined by flow cytometry using a Partec Cyflow cytometer. Data were analyzed by using the FlowJo software (FlowJo v10.3 for Windows; Treestar Inc, Ashland, OR, USA).

### Determination of caspase-1 activation

Neutrophils were stimulated as described above and at the specified times for each experiment, FAM-FLICA (FLICA) reagent was added according to manufacturer’s instructions. After additional culture, neutrophils were washed thoroughly, and analyzed for caspase-1 activation by flow cytometry. Alternatively, neutrophils were seeded in Cellview glass bottom dishes chambers (GBO) pre-treated with poly-L-lysine (0.01%), stimulated with LPS and two hours later, inflammasome activation was triggered by the addition of ATP (2.5 mM). The chamber was incubated at 37°C and 5% CO2 in the microscope incubator, and the FLICA probe was added ten minutes after ATP. Images were captured each 5 sec.

### Lactate dehydrogenase (LDH) Assay

Culture supernatants were harvested and LDH levels were measured by using a Pierce LDH Cytotoxicity Assay Kit according to manufacturer’s instructions. The same number of cells were lysed with 0.5% Triton X-100 and used as a positive control.

### Cytokine quantification

After neutrophil stimulation, cell supernatants were collected, and in the indicated cases, the cell pellets were treated with 0.1% Triton-X100 and protease inhibitors. Cytokines were quantified by ELISA following the manufacturer’s instructions.

### Automated image analysis

Images were analyzed using Fiji software and macros for automatized image quantification were designed as previously described (19). Regions of interest (ROIs) of cells were generated using the TRITC-phalloidin signal or the LC3B signal on the corresponding images. Briefly, a low threshold was applied for the creation of a mask image and individual ROIs were determined by size. For F-actin determination, the TRITC raw intensity density of the phalloidin signal was measured in each individual ROI. To quantify the degree of colocalization between LC3B and ASC, we performed an image analysis by using the Manders’ coefficients. The values of these coefficients range from 0 to 1, for no colocalization to perfect colocalization, respectively. For the colocalization of vesicular LC3B and ASC, a threshold was established and Manders’ M1 (LC3B) and M2 (ASC) coefficients of individual cells were determined using the coloc2 plugin.

### Whole cell extract and western blot analysis

Extracts from 5x10^6^ neutrophils were denatured in lysis buffer (Tris-HCl 60 mM, 1% SDS) with the addition of a 2.5X final concentration of protease inhibitors (cOmplete™, EDTA-free Protease Inhibitor Cocktail) for 10 min at 95°C. Then centrifuged at 13.000 rpm for 10 min at room temperature and supernatants were collected. Protein concentration was measured, and samples (65 μg per lane) were prepared with sample buffer 6X. Next, samples were separated in SDS-PAGE gel with 12% of polyacrylamide, and electrophoretically transferred onto polyvinylidene difluoride membranes (PVDF) membranes. Non-specific binding sites were blocked by soaking the membranes in Tris buffer saline (TBS), with 0.1% BSA, 0.4% Tween 20 and 1 mM EDTA for 1 hour at room temperature. The different proteins (as indicated in the figures and figure legends) were detected with specific primary antibodies and their corresponding horseradish peroxidase (HRP)-conjugated secondary antibodies followed by incubation and development with ECL. Note: Raw films of western blots are shown in **Supplementary Figure S10**.

### Statistical analysis

Statistical analysis was performed using GraphPad Prism 7 for Windows version 7.04, GraphPad Software, La Jolla, CA, USA or InfoStat software (Córdoba, Argentina) for Kruskall-Wallis analysis. Statistical significance was defined as p < 0.05.

## Results

### Human neutrophil treatment with the serine protease inhibitor AEBSF restrains autophagy impairing IL-1β secretion

**Figure 1:**
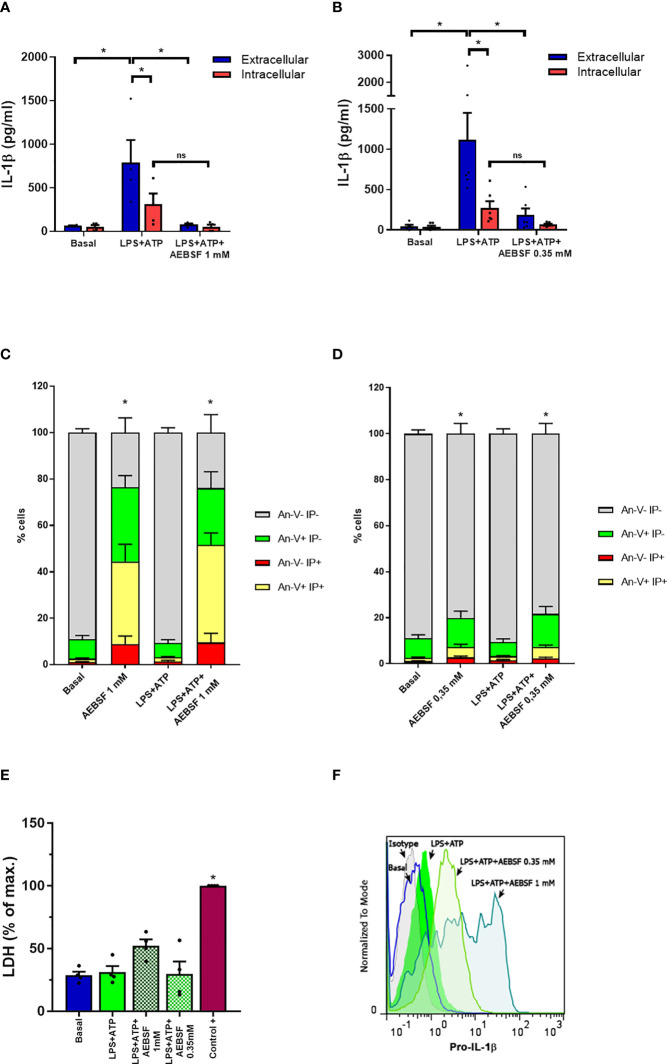
Treatment of neutrophils with the NSPs inhibitor AEBSF reduces IL-1β secretion without compromising pro-IL-1β synthesis. **(A, B)** Extracellular (blue bars) and intracellular (red bars) concentrations of IL-1β in culture supernatants of unstimulated (basal) or LPS + ATP-stimulated neutrophils for 5 h in the absence or presence of AEBSF added 30 min before LPS stimulation. Data represent the mean ± SEM of experiments performed in triplicate with 4 donors. **(C, D)** Apoptosis evaluated by annexin V-PI staining and flow cytometry in the cell pellets of the same donors evaluated in **(A)** and **(B)**. **(E)** LDH levels determined in culture supernatants of neutrophils treated as indicated and expressed as % of maximal LDH content, represented by the amount of LDH of a whole extract of the same number of neutrophils employed in the assay (control+). Data represent the mean ± SEM of experiments performed with 4 donors. **(F)** Pro-IL-1β expression in neutrophils stimulated or not with LPS+ATP (2.5 h p.s.) and pretreated or not with AEBSF (0.35 or 1 mM) evaluated by immunostaining and flow cytometry. Histograms are representative of experiments with 4 donors. *p<0.05. Two-way ANOVA followed by Bonferroni’s multiple comparisons test. ns, non-significant..

In a previous work, we determined that an autophagy-dependent mechanism is involved in IL-1β secretion in human neutrophils (19). In that study, we determined that neutrophil treatment with the NSPs inhibitor AEBSF (1 mM) added 1 h post-LPS-stimulation (p.s.) inhibited IL-1β secretion. This inhibitor did not induce the accumulation of intracellular mature IL-1β, although it tended to increase intracellular pro-IL-1β levels at 5 h p.s (19). We hypothesized that this was a consequence of a balance between two opposite effects of NSPs, on the one hand, their potential contribution to pro-IL-1β processing, and on the other one, their capacity to mediate IL-1β degradation inside the autolysosomes. However, NSPs might also modulate neutrophil IL-1β secretion by other pathways. To get insight into these other possibilities, we first performed assays to determine whether the addition of AEBSF before LPS stimulation reproduced the effects we observed when it was added later. Treatment of neutrophils with AEBSF (1 mM or 0.35 mM) 30 min before LPS stimulation nearly abrogated IL-1β secretion at 5 h p.s., a time point that according to our previous studies maximal IL-1β secretion is detected (**Figures 1A, B** **)**. Moreover, AEBSF also abolished intracellular IL-1β levels (**Figures 1A, B** **)**. These findings could not be attributed to cell death because even though AEBSF 1 mM induced a marked reduction in cell viability evaluated by either Annexin V and propidium iodide (PI) staining (**Figures 1C, D** and **Supplementary Figure S1**) and LDH release (**Figure 1E**), most of the cells were viable when treated with AEBSF 0.35 mM. Furthermore, AEBSF did not inhibit pro-IL-1β synthesis as indicated by flow cytometry assays using a specific antibody that recognizes this precursor (**Figure 1F**).

**Figure 2:**
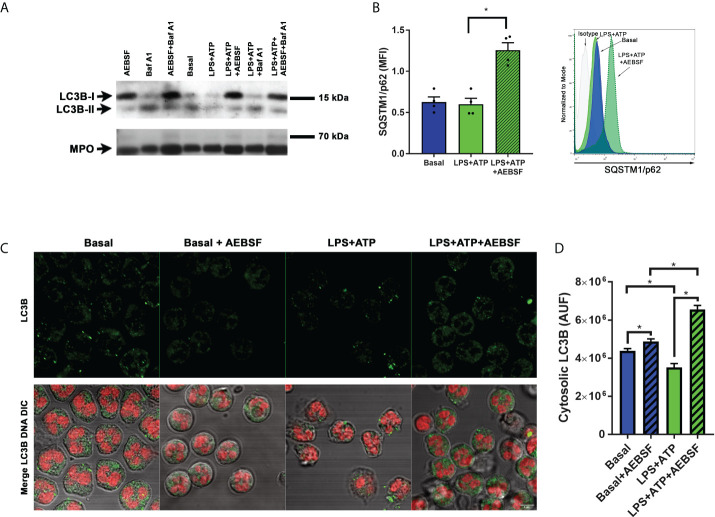
Treatment of neutrophils with AEBSF inhibits autophagy induction. **(A)** Immunoblot analysis of LC3B in lysates of human neutrophils pre-treated or not with AEBSF (0.35 mM; t= -30 min), then stimulated or not with LPS (t= 0); treated or not with the autophagy flux blocker Baf A1 (t= 1 h), and with ATP (t= 2 h), assessed at 2 h and 15 min post-LPS stimulation. Immunoblot is representative of 2 independent experiments. Myeloperoxidase (MPO) was employed as loading control. **(B)** Median fluorescence intensity (MFI) levels of the autophagy receptor p62/SQSTM1 evaluated by immunostaining and flow cytometry at 2 h and 15 min post-LPS stimulation. Data represent the mean ± SEM of experiments performed with 4 donors (left) and representative histograms of one of these experiments (right). *p < 0.05. One-way ANOVA followed by Holm-Sidak’s multiple comparisons test. **(C)** Representative CLSM images of neutrophils pre-treated or not with AEBSF and stimulated or not with LPS+ATP for 4 h. Cells were stained with a specific antibody anti-LC3B and the corresponding secondary antibody and DNA was stained with ToPro-3. Images are representative of experiments with 3 different donors. **(D)** Quantification of the images of experiments performed with 3 donors as shown in **(C)** Data represent the fluorescence corresponding to cytosolic LC3B expressed in arbitrary units of fluorescence (AUF). *p<0.05. Kruskal-Wallis test.

Considering that IL-1β is exported from human neutrophils by an autophagy-dependent mechanism (19), we first evaluated the impact of AEBSF on the autophagic pathway. To this aim, we took advantage of the fact that in cells undergoing autophagy, the cytoplasmic protein LC3B-I conjugates with phosphatidylethanolamine in the membrane of autophagosomes (LC3B-II) and both isoforms can be identified by western blot. Thus, we treated or not human neutrophils with AEBSF and stimulated them or not with LPS and ATP as we previously showed that these stimuli induce the neutrophil autophagy flux. As a control, we employed the autolysosomal degradation blocker Bafilomycin A1 (Baf A1) to determine the effect of the inhibition of autophagy flux in these cells. As depicted in **Figure 2A**, inhibition of NSPs with AEBSF induced a strong increase in the LC3B-I band in both unstimulated and LPS+ATP stimulated cells. As expected, treatment with Baf A1 increased the LC3B-II isoform especially in LPS+ATP-stimulated neutrophils. But more importantly, the combined treatment of AEBSF and Baf A1 did not increase LC3B-II expression compared to Baf A1 treatment alone, suggesting that AEBSF inhibits autophagy induction but does not block the autophagy flux.

We also evaluated the expression of SQSTM1/p62, a protein that links LC3 with ubiquitinated substrates to incorporate them into autophagosomes for degradation. Thus, inhibition of autophagy induction usually correlates with increased SQSTM1/p62 levels. Results from flow cytometry studies indicated that inhibition of NSPs markedly increased SQSTM1/p62 levels (**Figure 2B**). In assays performed by immunostaining and confocal microscopy, we also detected an accumulation of cytosolic LC3B when LPS-stimulated cells were treated with AEBSF (**Figures 2C, D**). Altogether, our findings suggest that NSPs inhibition modulates the neutrophil autophagy process. Thus, the ability of AEBSF to reduce neutrophil IL-1β secretion could be due to its capacity to impair IL-1β entry into autophagy vesicles.

### Autophagy and NSPs modulate caspase-1 activity

**Figure 3:**
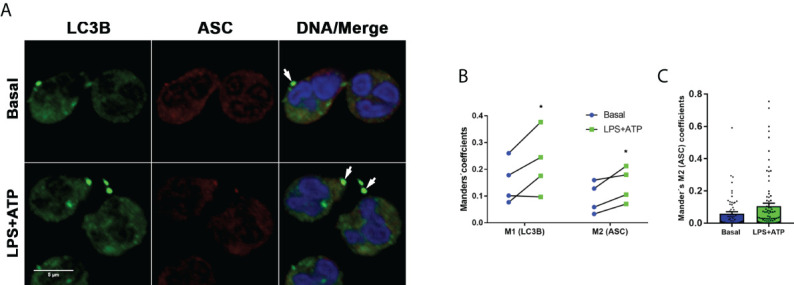
Inflammasomes are targeted to autophagosomes. **(A)** Representative CLSM images of neutrophils stimulated or not with LPS and 2 hours later treated or not with ATP. At 4 h post-LPS stimulation, cells were fixed, permeabilized and stained with specific antibodies anti-LC3B (green) and ASC (red). Images are representative of experiments with 4 different donors. **(B)** Manders´ coefficients (M1; the amount of vesicular LC3B that colocalizes with ASC) and M2 (the amount of ASC that colocalizes with LC3B) calculated by image quantification of experiments performed with 4 donors as in **(A)** in which at least 60 cells were analyzed for each condition by using a specific macro with Fiji software. Each data in the graph corresponds to the mean of Manders´ coefficients of at least 60 cells of each individual donor per experimental condition. *p<0.05. Kruskal-Wallis test. **(C)** Data from a representative experiment of those included in the graph depicted in B, from which the mean value of the Mander’s M2 coefficient for LC3B was calculated.

As determined in our previous studies, and as confirmed here by time-lapse microscopy (**Supplementary video 1**), neutrophil stimulation with LPS+ATP induces caspase-1 activation, and its inhibition significantly reduces neutrophil IL-1β secretion (18). Caspase-1 is activated within the inflammasomes and prior reports in macrophages indicated that autophagy engulfs inflammasomes and mediates their degradation (21). Thus, we reasoned that AEBSF by inhibiting autophagy might modulate the stability of active caspase-1. Therefore, we first determined if inflammasomes in human neutrophils are also targeted to autophagosomes. Supporting this possibility, the ASC adaptor molecule could be found colocalizing with LC3B puncta by immunostaining and CLSM, especially when cells were stimulated with LPS+ATP (**Figures 3A–C**).

**Figure 4:**
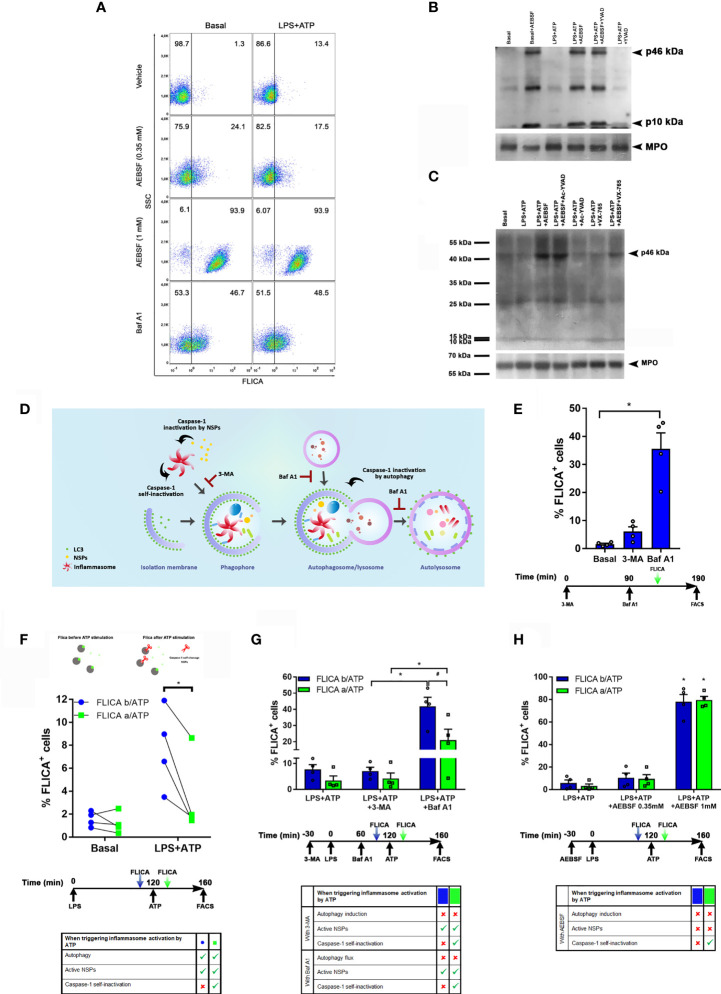
Caspase-1 activity is regulated by autophagy, self-inactivation and by NSPs. **(A)** Neutrophils were pre-treated or not with AEBSF (t= -30 min), then stimulated or not with LPS (t= 0); and treated or not with Baf A1 (t= 1 h). At 1 h 50 min the FLICA probe was added and inflammasome activation was stimulated by ATP at 2 h and cells were assessed at 2 h 40 min by flow cytometry. Dot plots are representative of experiments with 4 donors. (B and C) Immunoblot analysis of caspase-1 in lysates of human neutrophils pre-treated or not with AEBSF (0.35 mM), Ac-YVAD-CMK (50 μM), VX-765 (50 µM) or AEBSF and the corresponding caspase-1 inhibitor together, at t= -30 min, then stimulated or not with LPS (t=0) and ATP (t= 2 h) and assessed at 3 h 30 min post-LPS stimulation. MPO levels are shown as loading control. Immunoblots are representative of 3 **(B)** and 2 **(C)** independent experiments. **(D)** Rationale model to explain how the different inhibitors would impact on caspase-1 activity and its detection. **(E)** Neutrophils were treated or not with 3-MA or Baf A1 as indicated in the schedule below the graph, stained with the FLICA probe and cell fluorescence was determined by flow cytometry. **(F–H)** Neutrophils were treated as indicated in the schedule below each graph. Where indicated the FLICA probe was added 10 min before (b/ATP; blue symbols/bars) or 10 min after (a/ATP; green symbols/bars) inflammasome activation by ATP, and fluorescence was determined by flow cytometry. (F; top part) depicts the rationale of the assay; grey: caspase-1, green: FLICA probe, red scissors: proteolytic cleavage. *p<0.05 **(D)** One way ANOVA followed by Sidak’s multiple comparisons test; **(E)** Paired t test; **(F–G)** Two-way ANOVA followed by Tukey’s multiple comparisons test. #p<0.05 **(F)** Two-way ANOVA followed by Sidak’s multiple comparisons test.

Hence, subsequently, we determined the effect of AEBSF on caspase-1 by employing FLICA, a fluorescent probe that irreversibly binds to active caspase-1. As expected, stimulation of neutrophils with LPS+ATP increased the percentage of cells with active caspase-1. Treatment with AEBSF considerably increased this number further (**Figure 4A**). Intriguingly, we detected a similar percentage of cells with active caspase-1 in either unstimulated (**Figure 4A** left panels) or LPS+ATP-stimulated neutrophils (**Figure 4A** right panels) when treated with AEBSF. These findings suggested that inhibition of either autophagy or NSPs conducted to an increase in the active caspase-1 levels independently of the cellular stimulation status. Since treatment of either unstimulated- or LPS+ATP-stimulated neutrophils with the autophagy flux blocking agent Baf A1 also markedly increased the percentage of cells with active caspase-1 (**Figure 4A**; lower panels), our results suggest that inhibition of neutrophil autophagy leads to caspase-1 activation. These findings are in agreement with previous observations in macrophages stimulated with LPS+ATP in which the deficiency in the autophagic proteins LC3B and beclin 1 enhanced the activation of caspase-1 (22). However, it is important to note that our findings do not rule out that part of the active caspase-1 we detected was also due to an increase in its stability because it can no longer be degraded inside autophagosomes (see below in **Figure 4G**).

In western blot assays performed using an antibody raised against the C-terminus of the human caspase-1 (p10 subunit), we detected a 10 kDa band which represents a post-activation cleavage product of this enzyme, either in unstimulated or LPS+ATP-stimulated neutrophils (**Figure 4B**), although in the last case, a much more intense band was detected. These results suggest that some caspase-1 might be found constitutively active in neutrophils. In agreement with results obtained by FLICA staining, treatment of neutrophils with AEBSF elicited an increased p10 band confirming that inhibition of autophagy led to caspase-1 activation.

Noteworthy, treatment with AEBSF also induced the appearance of the p46 kDa isoform. Previous studies showed that the dominant species of active caspase-1 elicited by inflammasomes consists of dimers of the full-length p46 and a transient species, p33/p10 (14). That work indicated that further p33/p10 auto-processing releases p20/p10 from the inflammasome, whereupon the tetramer becomes unstable in cells and protease activity is terminated. According to these findings, treatment with Ac-YVAD-CMK, which irreversibly bind to active caspase-1, should lead to the stabilization of the dimers of p46 isoform (and the p33/10 isoform) on the inflammasome since it would avoid caspase-1 self-inactivating cleavage. However, when we treated human neutrophils with Ac-YVAD-CMK, we were unable to detect an accumulation of the p46 isoform and the levels of the p10 band were similar to those observed upon LPS+ATP stimulation alone (**Figure 4B**). These results suggest that in contrast to what happens in macrophages, when the neutrophil’s caspase-1 is irreversibly inhibited by Ac-YVAD-CMK, it can still be cleaved by other proteases. In fact, with this antibody (sc-515) we only detected the p46 kDa isoform upon treatment with AEBSF, the condition that, according to FLICA assays, increases the percentage of cells with active caspase-1, and upon simultaneous treatment with AEBSF and Ac-YVAD-CMK (**Figure 4B**). We surmise that AEBSF avoided active caspase-1 cleavage, increasing its intracellular concentration in such a way that it exceeded the threshold limit of detection by the antibody. However, as the full-length p46 isoform is expected to be present even in its monomeric (inactive) state under basal conditions, we repeated the assays by employing another anti-caspase-1 antibody (sc-56036). This antibody was more effective in recognizing the caspase-1 precursor, allowing us to detect the p46 kDa band under all the conditions evaluated even in unstimulated (basal) cells (**Figure 4C**). Of mention, in these new set of assays, we also examined the effect of VX-765, a caspase-1/4 inhibitor either alone or together with AEBSF. As observed with Ac-YVAD-CMK, inhibition of caspase-1/4 with VX-765 did not increased full-length caspase-1, something that was observed when it was added together with AEBSF (**Figure 4C**).

These results, together with those of FLICA assays, suggest that the inhibition of autophagy elicits inflammasome activation, and that inhibition of NSPs either directly or by its effect on autophagy, might control the stability of the active caspase-1 isoform. Thus, we then compared caspase-1 activation levels by FLICA staining in neutrophils in which autophagy was inhibited by 3-MA and Baf A1. We reasoned that as 3-MA inhibits autophagy induction, even though it leads to inflammasome activation, it would impair its targeting to the autophagosomes. Thus, after activation, caspase-1 might self-inactivate or eventually be a target of cytosolic NSPs. By contrast, as Baf A1 inhibits the autophagy flux avoiding autolysosome degradation, even if Baf A1 could induce inflammasome activation, this complex might still be targeted to the autophagosome. Thus, under these conditions, caspase-1 might undergo self-inactivation, but it would be protected from the action of cytosolic NSPs (**Figure 4D**). As shown in **Figure 4E**, both autophagy inhibitors increased the percentage of cells with active caspase-1 with respect to basal conditions. However, in accordance with our presumption, these levels were higher upon treatment with Baf A1. These findings suggest that active caspase-1 is susceptible to inactivation in the cytosol.

Afterwards, we performed assays in which we added to LPS+ATP-stimulated neutrophils the FLICA probe either 10 min before or after inflammasome activation by ATP. Since this probe rapidly diffuses inside the cell and irreversibly binds to and disables active caspase-1, we reasoned that if it is added before inflammasome activation, it would hamper the potential caspase-1 self-inactivation. By contrast, when it is added after ATP, a portion of caspase-1 might self-inactivate before FLICA has the chance to bind to its active-site (**Figure 4F** top). In fact, we detected higher levels of active caspase-1 when FLICA was added before ATP treatment (**Figure 4F**; bottom), suggesting that, as previously reported, caspase-1 can undergo self-inactivation.

We also reasoned that as 3-MA inhibits autophagy induction, upon treatment of neutrophils with this inhibitor, caspase-1 not only might undergo self-inactivation but also be more susceptible to inactivation by cytosolic NSPs. Also, upon treatment with Baf A1, caspase-1 might remain active longer because it could still be incorporated in autophagosomes but not degraded. Thus, the comparison of FLICA signal upon treatment with both inhibitors might provide information of the contribution of autophagy to caspase-1 inactivation. Moreover, by adding FLICA before and after ATP stimulation we could also obtain an estimation of the level of caspase-1 self-inactivation under these conditions. Results in **Figure 4G** showed a higher percentage of FLICA positive cells when neutrophils were treated with Baf A1 compared to those treated with 3-MA, suggesting that autophagy markedly contributes to active caspase-1 removal. Moreover, we detected lower levels of cells with active caspase-1 when FLICA was added after ATP stimulation under all the conditions evaluated (**Figure 4G**), suggesting that caspase-1 self-inactivation also takes place rapidly after activation as determined by Schroder’s group. Of note, we observed similar levels of cells with active caspase-1 even if they were treated or not with 3-MA, suggesting that if caspase-1 is not incorporated into autophagosomes it can be rapidly inactivated in the cytosol.

Finally, we performed additional assays with LPS+ATP-stimulated neutrophils in which we evaluated caspase-1 activation by adding FLICA either before or after ATP treatment in the presence or absence of AEBSF (**Figure 4H**). AEBSF augmented the percentage of cells with active caspase-1 but this increase was independent of the moment at which the FLICA probe was added. These results suggest that when autophagy induction is inhibited, mainly NSPs contribute to caspase-1 inactivation.

**Figure 5:**
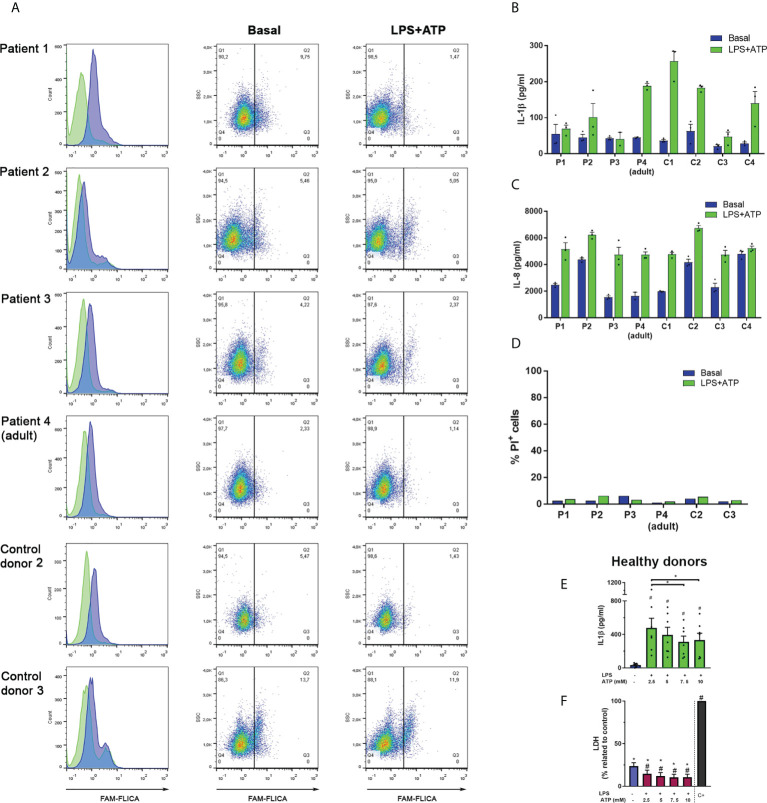
Responses of neutrophils isolated from patients who are carriers of the GOF *NLRP3* c.1322C>T (p.A441V) mutation. **(A)** Neutrophils from patients and aged-matched control donors were stimulated or not with LPS (t= 0) and 2 h later were stimulated or not with ATP. Ten min later, the FLICA probe was added, and cell fluorescence was determined at 2 h 40 min by flow cytometry. Data are depicted as histograms (blue, basal -unstimulated- condition; green, LPS+ATP-stimulated cells) to appreciate FLICA signal reduction upon stimulation; and dot plots, to show the % of cells with FLICA signal higher than the same arbitrary threshold set for all donors. **(B–D)** Neutrophils from patients and aged-matched control donors were stimulated or not with LPS (t= 0) and 2 h later were stimulated or not with ATP. At 5 h post-LPS stimulation IL-1β **(B)** or IL-8 **(C)** concentrations in culture supernatants were determined by ELISA and PI incorporation in the cell pellets was evaluated by flow cytometry **(D)**. In **(B)** and **(C)**, each bar represents the mean+SEM of the cytokine concentrations of an individual donor evaluated in triplicate. **(E**, **F)** Neutrophils from healthy donors were treated or not with LPS, and 2 h later with the indicated ATP concentrations. At 5 h post-LPS stimulation IL-1β **(E)** or LDH activity **(F)** were determined. Two-way ANOVA followed by Tukey’s multiple comparisons test. # and *p < 0.05. # vs basal **(E, F)**; * vs positive control **(F)**.

Caspase-1 inactivation was also evident in studies performed with cells from pediatric patients with a GOF *NLRP3*-mutation (**Figure 5A**). In those neutrophils, we could detect some constitutive active caspase-1 although we also found activation in age-matched control neutrophils. However, when we stimulated cells with LPS+ATP and added FLICA 10 min after inflammasome activation by ATP, we not only detected a reduction in the percentage of cells with active caspase-1, but also, a decrease in FLICA fluorescence intensity in the whole cell population. A similar behavior was observed in neutrophils from the patients’ mother (patient 4) who is also carrier of the *NLRP3* mutation even though the constitutive caspase-1 activation level was lower than in her offspring’s. In neutrophils of the aged-matched control donors a decrease in FLICA fluorescence intensity was also observed after LPS+ATP stimulation as compared to that in unstimulated neutrophils, although the reduction was not as pronounced as in patients’ neutrophils (**Figure 5A**).

Comparison of the median FLICA fluorescence intensity of unstimulated neutrophils with LPS+ATP-stimulated neutrophils of adult healthy donors when FLICA was added 10 min after ATP stimulation, also showed a reduction in active caspase-1 in 8 out of 14 donors (**Supplementary Figure S2**).

Altogether these results suggest that after inflammasome activation, caspase-1 substantially undergoes rapid deactivation by NSPs, autophagy and self-inactivation.

### Caspase-1 activation is necessary but not sufficient for neutrophil IL-1β secretion.

Intriguingly, despite the increased constitutive activity of caspase-1 observed in the neutrophils from some patients carrying a GOF-*NLRP3* mutation and in control donor 3, these cells did not exhibit an augmented IL-1β secretion either basally or when they were stimulated with LPS+ATP (**Figure 5B**) or LPS alone (**Supplementary Figure S3**). However, the secretion of IL-8, a cytokine that does not involve caspase-1 processing, was similar among control donors and patients’ neutrophils (**Figure 5C**). In fact, patients’ neutrophils secreted lower IL-1β amounts when stimulated by LPS+ATP than those released by neutrophils from most age-matched control donors, although their monocytes exhibited an increased IL-1β release (**Supplementary Figure S4**). The differences in the capacity to secrete IL-1β were not due to cell death, as neutrophils from patients’ and control donors incorporated low and similar levels of PI (**Figure 5D**). Noteworthy, the neutrophils from the patients’ mother (patient 4) which carry the same *NLRP3* mutation, did not show differences in IL-1β secretion regarding most control donors and were still able to respond to LPS+ATP stimulation. However, her neutrophils showed low levels of constitutive caspase-1 activation. By contrast, control donor 3 (C3) neutrophils, which showed a remarkable high level of constitutive caspase-1 activation, exhibited a reduced capacity to release IL-1β.

Our previous findings (18), confirmed in this work (**Supplementary Figure S5**) indicated that inhibition of caspase-1 with either Ac-YVAD-CMK or with VX-765 constrains IL-1β secretion. Thus, these findings together with results of this study suggest that caspase-1 activation is necessary for neutrophil IL-1β secretion, but its increased activation might be detrimental to this end. In agreement with this possibility, IL-1β secretion from healthy donors’ neutrophils stimulated with LPS, was reduced upon treatment with increasing concentrations of ATP (**Figure 5E**). Of note, this reduction was not due to cell death (**Figure 5F**).

One of the causes underlying these observations might be that a greater caspase-1 activity could lead to an increase in cytosolic NSPs that might contribute not only to process, but also to degrade pro-IL-1β. In fact, previous studies showed that in neutrophils, active caspase-1 cleaves Gasdermin D (GSDMD) generating N-GSDMD that polymerizes on the membrane of azurophil granules and causes leakage of NE into the cytosol (10). As the human pro-IL-1β sequence has many NE and other NSPs cleavage target sites [**Supplementary Figure S6** and (23)], we speculated that after caspase-1 activation and N-GSDMD polymerization on the azurophil granules membranes, NSPs might be released to the cytosol not only being able to inactivate caspase-1 (as we showed above) but also process and/or degrade pro-IL-1β.

**Figure 6:**
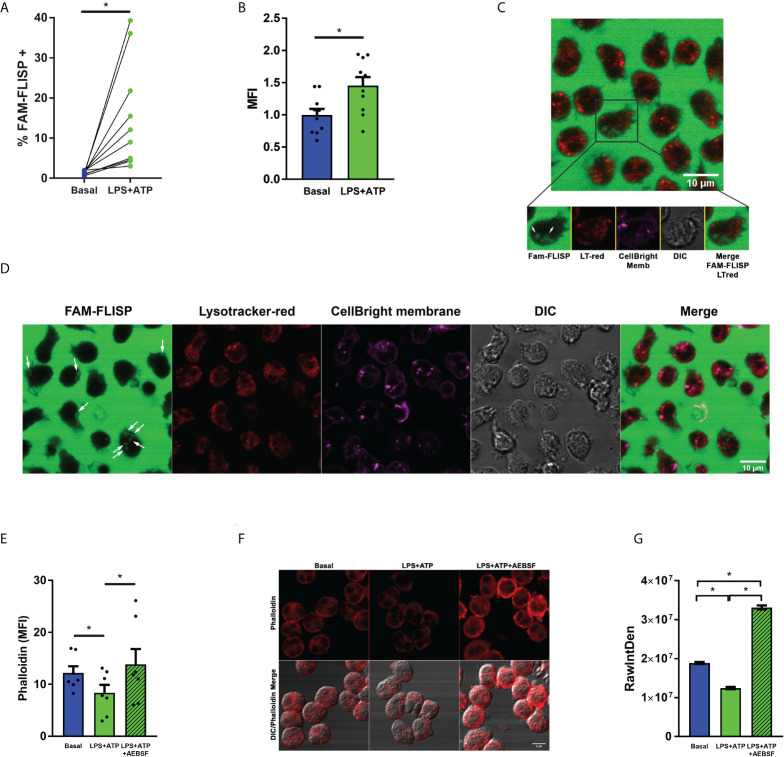
Neutrophil serine proteases undergo activation upon LPS+ATP stimulation. **(A, B)** Neutrophils from healthy donors were stimulated or not with LPS (t= 0), stained with the fluorescent probe FAM-FLISP (t= 115 min), stimulated or not with ATP (t= 120 min) and fluorescence was recorded by flow cytometry (t= 155 min). **(A)** Each point depicts the % of cells with FAM-FLISP positive signal. Results corresponding to each donor are linked with a line. **(B)** Mean ± SEM of the median fluorescence intensity (MFI) values of FAM-FLICA signal. *p<0.05 One-tailed Paired t test. **(C, D)** Representative images acquired by time-lapse confocal microscopy of neutrophils stained with CellBrite fix640 to delimit cell contour (magenta) and the fluorescent probes FAM‐FLISP to detect active NSPs (green) and Lysotracker red (red). Cells were stimulated with LPS (1 μg/ml in C or 150 pg/ml in D) and treated with ATP (2.5 mM; t= 2h). Images were acquired every 2 min **(C)** and 63 sec **(D)**. White arrows show the diffuse pattern of active NSPs. Images are representative of 3 **(C)** and 1 **(D)** experiments. Of note, the intense green fluorescence outside the cells is emitted by the probe that had to be present during the whole experiment to increase the sensitivity of the assay. **(E–G)** Actin dynamic evaluated as a surrogate marker of NSP enzymatic activity in the cytosol. Neutrophils were pre-treated or not with AEBSF (0.35 mM), stimulated or not with LPS and 2 h later treated or not with ATP. Then, cells were fixed, permeabilized and stained with TRITC-Phalloidin. Cell fluorescence was evaluated by flow cytometry **(E)** and images were acquired by CLSM **(F)** and quantitated **(G)**. TRITC raw intensity density of the phalloidin signal was measured for each individual cell. **(G)** Graph depicts the mean ± SEM of raw intensity density values of 3 independent experiments in which at least 63 cells were analyzed. *p<0.05 One-way ANOVA **(D)** and Kruskal-Wallis test **(F)**.

To get insight in this issue, we first analyzed if LPS+ATP stimulation induces NSPs activation by employing the fluorescent probe FAM-FLISP that binds to active serine proteases. We confirmed its activation by flow cytometry upon LPS+ATP treatment (**Figures 6A, B**). Then, we determined if under our experimental conditions, NSPs are released in an active state into the cytosol by employing FAM-FLISP and analyzing its cell distribution by time-lapse CLSM. In these assays we also employed Lysotracker red to discriminate the acidic compartments, among them azurophil granules. **Figures 6C, D**, and **Supplementary video 2, 3** show that upon LPS+ATP stimulation active NSPs are not found in the acidic granular compartments. However, a diffuse signal of active NSPs was detected in these cells, consistent with the notion that they were in the cytosol.

This possibility was further supported by results of additional assays that evaluated F-actin levels, since previous studies showed that when NE is released to the cytosol it binds and degrades F-actin (9). By employing phalloidin staining and flow cytometry, we detected a reduction of phalloidin fluorescence levels in LPS+ATP-stimulated neutrophils indicating, as expected, a promotion of the actin dynamics (**Figure 6E**). By contrast, inhibition of NSPs with AEBSF markedly increased phalloidin fluorescence over that observed upon LPS+ATP stimulation (**Figure 6E**). Similar findings were made when phalloidin staining was evaluated by CLSM (**Figures 6F, G**).

Altogether, these results support that neutrophil stimulation with LPS+ATP induces active NSPs leakage to the cytosol where they might contribute to inactivate the caspase-1 and to either process or eventually cleave/degrade pro-IL-1β.

**Figure 7:**
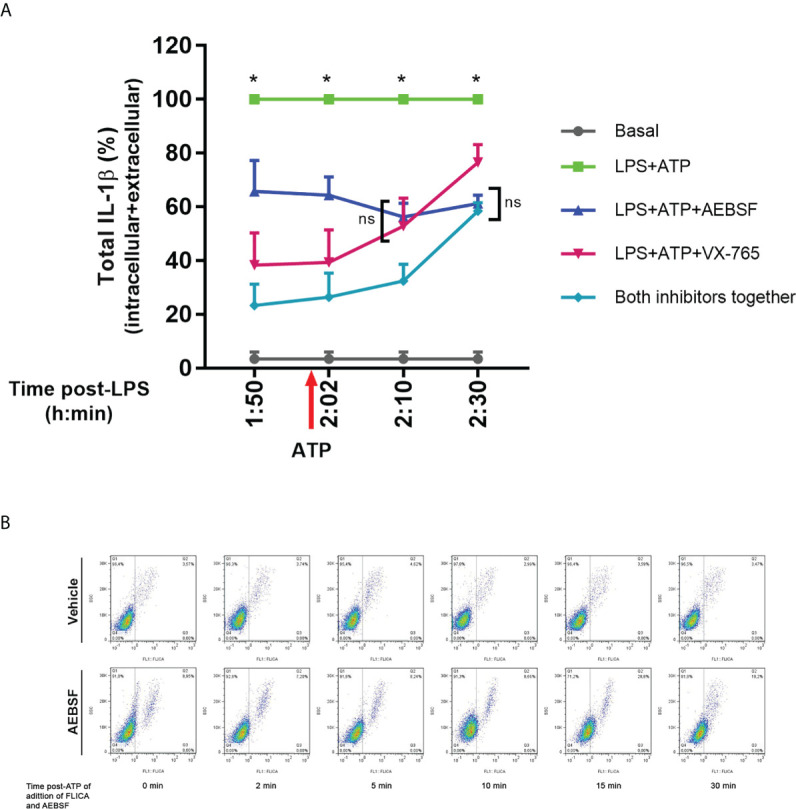
Dynamic of pro-IL-1β processing by caspase-1 and NSPs. **(A)** Total (intracellular+extracellular) mature IL-1β levels determined by ELISA in neutrophils stimulated with LPS+ATP either in the presence of AEBSF, VX-765 (caspase-1/4 inhibitor) and both inhibitors together, added at different time points before and after inflammasome activation with ATP. Neutrophils were stimulated or not with LPS and 2 h later with ATP. Ten min before ATP treatment or 2, 10 or 30 min after, cells were treated with AEBSF (0.35 mM), VX-765 (50 µM) or both inhibitors together. At 5 h post-LPS stimulation, total (intracellular+extracellular) concentrations of mature IL-1β were determined by ELISA. Data are depicted as % of the levels of mature IL-1β produced upon LPS+ATP stimulation alone and represent the mean ± SEM of experiments performed in duplicate with 4 donors. *p<0.05 LPS+ATP vs each treatment at the corresponding time point; ns: non-significant; Two-way ANOVA with Tukey’s multiple comparisons test. **(B)** Effect of inhibition of NSPs at different time points after inflammasome activation with ATP on caspase-1 activity. Neutrophils were stimulated with LPS for 2 h, and then were treated with ATP to induce inflammasome activation. At the time points post ATP-addition indicated below each dot plot, FLICA was added or not simultaneously with AEBSF to trap during a 5 minutes-lapse all active caspase-1. Images depict representative dot plots of experiments performed with 4 donors showing FLICA fluorescence of neutrophils after having excluded the doublets.

To obtain further evidence of these possibilities, we stimulated neutrophils with LPS, and before or after the addition of ATP, we treated them either with AEBSF, VX-765 or both inhibitors together. At the end of the culture, we determined pro-IL-1β processing by evaluating the total levels (intracellular + extracellular) of mature IL-1β (**Figure 7A**). We found that both, AEBSF and VX-765 partially inhibited IL-1β processing at all the time points they were added. However, we detected greater IL-1β processing (higher levels of mature IL-1β) when NSPs were inhibited either 10 min before or 2 min after inflammasome activation with ATP, than when caspase-1 was inhibited with VX-765 at the same time points, suggesting that immediately after inflammasome activation the caspase-1 exerts a major role in pro-IL-1β processing. However, when the caspase-1 inhibitor was added 10 min after the inflammasome activation, it had a lower impact in IL-1β processing, but if NSPs were simultaneously inhibited by AEBSF, IL-1β processing was markedly reduced. These results suggest that caspase-1 was less relevant for IL-1β processing at this time point, in agreement with the possibility that part of the enzyme had undergone inactivation. The fact that both inhibitors added together reduced even more the processing of the cytokine than caspase-1 inhibition alone, confirms that NSPs also play a role in IL-1β processing at this moment. The results we obtained when inhibitors were added at 30 min post-ATP indicating that caspase-1 inhibition reduced even less IL-1β processing in contrast to NSPs inhibition, suggested that by this time NSPs play a major role in the processing of the cytokine.

Furthermore, to get insight if NSPs are involved in the caspase-1 activity reduction that we observed at 10 min after inflammasome activation, we performed additional assays with the FLICA probe (**Figure 7B**). In these assays, we added or not AEBSF simultaneously with FLICA at different time points post-inflammasome activation with ATP and incubated them during a 5 minutes-lapse to allow FLICA to bind all active caspase-1 that could be present in this time frame. We observed a reduction in FLICA signal at 10 min post-ATP in the absence of the inhibitor (top panel), while the addition of AEBSF increased active caspase-1 levels. Moreover, the addition of AEBSF led to a maximal increase in active caspase-1 levels when it was added at 15 min post-ATP, but these values were reduced again when AEBSF was added at 30 min post-ATP. These results are in agreement with the possibility that by this time-point part of the caspase-1 had undergone inactivation being unable to be trapped by FLICA. Considering that in these assays we only analyzed the FLICA signal that accumulated in a 5-min lapse time, and AEBSF was acting only during this period, these results probably reflect the effects of the NSPs inhibition on caspase-1 instead of the contribution of AEBSF to autophagy inhibition.

### NSPs play a major role in pro-IL-1β processing

**Figure 8:**
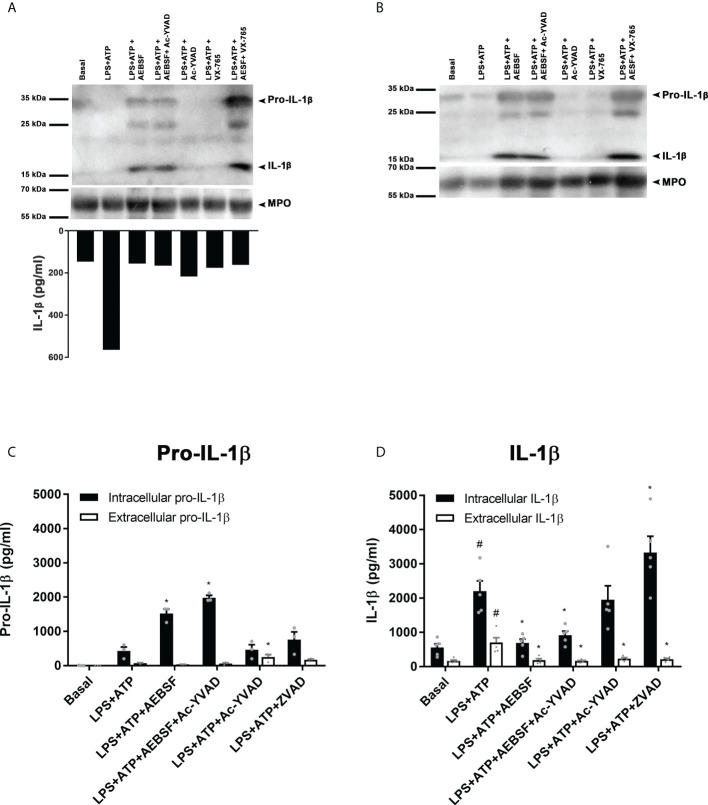
NSPs but not caspase-1 play a major role in pro-IL-1β processing. (**A** upper part and **B**) Neutrophils were pre-treated for 30 min in the presence or absence of AEBSF (1 mM), Ac-YVAD-CMK (50 µM), VX-765 (50 µM) or their combination of AEBSF and one of the caspase-1 inhibitors, then cultured with or without LPS for 2 h and after were stimulated or not with ATP (2.5 mM) for another 1.5 h. Then, whole cell extracts were subjected to Western blot **(A, B)** and supernatants collected for ELISA quantification (A; bottom part). Western blots in A and B belong to the same cells extracts that were electrophoresed and electro-transferred twice, one probed with an anti-human IL-1β antibody from Santa Cruz (antibody H-153; A) and the other one with a Novus Biological antibody (NB600-633; B). (**A**, upper part and **B**) Representative Western blot assay (n=6; **A** and n=2; **B**) of pro-IL-1β and mature IL-1β expression; and (A, bottom part) IL-1β concentration in culture supernatants of the same cells from which lysates were immunoblotted in **(A)** and **(B)**. **(C, D)** Concentrations determined by specific ELISAs of pro-IL-1β **(C)** and IL-1β **(D)** in culture supernatants and cell pellets of neutrophils stimulated in the absence or presence of AEBSF (0.35 mM), Ac-YVAD-CMK (50 µM), the combination of AEBSF and Ac-YVAD-CMK, or Z-VAD-FMK (50 µM) added 30 min before LPS stimulation, then stimulated or not for 2 h with LPS and then treated or not with ATP for another 1.5 h. Data represent the mean ± SEM of experiments with 3 **(C)** or 5 **(D)** donors performed in duplicate. *p<0.05 vs LPS+ATP; #p<0.05 vs Basal. One-way ANOVA with Sidak´s multiple comparisons test.

On the other hand, to further examine if NSPs contribute to process pro-IL-1β, we reasoned that AEBSF, that when added 30 min before LPS inhibited the induction of neutrophil autophagy, should impair IL-1β to enter autophagy vesicles and its secretion. Thus, by employing this compound we would be able to study in more depth the impact of NSPs and caspase-1 on the cytoplasmic processing of pro-IL-1β. To this end, we pre-treated LPS+ATP-stimulated neutrophils with AEBSF, Ac-YVAD-CMK, VX-765 or the combination of AEBSF with either caspase-1 inhibitors to compare their effects on pro-IL-1β expression and its processing in whole cell lysates by western blot at 3.5 h post-LPS stimulation (**Figure 8A** upper part and **8B**) and in the supernatants of the same cells by quantitation of IL-1β concentrations by ELISA (**Figure 8A** bottom part). Of mention, the same extracts were probed with two different anti-IL-1β antibodies to confirm the results (**Figure 8A** upper part and **8B**). As expected, and according to our previous studies (18), in cells stimulated with LPS+ATP we barely detected the pro-IL-1β band and most of the mature IL-1β was found in the culture supernatant (**Figure 8A** bottom part). Treatment with AEBSF alone, or in combination with Ac-YVAD-CMK or VX-765, markedly increased the band corresponding to pro-IL-1β. By contrast, treatment only with the caspase-1 inhibitors did not markedly modulate the pro-IL-1β and mature IL-1β bands compared to those observed with LPS+ATP alone as would be expected if caspase-1 plays a major role in processing this cytokine. However, both AEBSF and the caspase-1 inhibitors inhibited IL-1β release (**Figure 8A** bottom part) as we have already described for Ac-YVAD-CMK (18, 19) and confirmed in this work (**Supplementary Figure S5**). These assays indicated that pro-IL-1β accumulated not only when NSPs were inhibited and caspase-1 was expected to be active for a longer time (AEBSF condition; see **Figure 4A**), but also when both NSPs and caspase-1 were expected to be inactive (AEBSF+Ac-YVAD-CMK or AEBSF+VX-765 conditions). Besides, we did not detect extracellular IL-1β when LPS+ATP-stimulated cells were incubated with these inhibitors, even though cells in their absence secreted huge amounts of IL-1β (**Figure 8A** bottom part).

Of mention, due to differences in IL-1β secretion dynamics, in some donors we could detect a band corresponding to mature IL-1β upon LPS+ATP stimulation (**Supplementary Figure S7**) because it was not totally released at the time point analyzed. In these cases, we also detected mature IL-1β upon Ac-YVAD-CMK treatment, confirming that even when caspase-1 was inhibited IL-1β could still be processed. Altogether, these findings suggest that NSPs mainly contribute to pro-IL-1β processing in human neutrophils.

Finally, we performed additional assays to quantitate by specific ELISAs the intracellular and extracellular pro-IL-1β and IL-1β levels upon treatment with the inhibitors (**Figures 8C, D**). In agreement with western blot results, treatment with AEBSF alone or together with Ac-YVAD-CMK markedly increased the intracellular levels of pro-IL-1β (**Figure 8C**). Treatment with Ac-YVAD-CMK did not lead to pro-IL-1β accumulation. A similar behavior was detected by intracellular immunostaining and flow cytometry (**Supplementary Figure S8** **).** Furthermore, inhibition of caspase-1 neither modulated mature intracellular IL-1β levels as compared to those observed with LPS+ATP (**Figure 8D**). To determine if another caspase might overcome the absence of active caspase-1 in cells treated with Ac-YVAD-CMK, we evaluated the impact of the pan-caspase inhibitor Z-VAD-FMK (ZVAD) on pro-IL-1β processing and IL-1β secretion. However, this inhibitor increased further pro-IL-1β processing, rising the intracellular levels of mature IL-1β, even though it did not promote its secretion, ruling out that possibility. As expected, according to our previous results (18, 19), treatment with either AEBSF or Ac-YVAD-CMK did not induce IL-1β secretion, suggesting that even though caspase-1 does not play a major role in pro-IL-1β processing it is still required for IL-1β secretion. Of mention, an elastase inhibitor, AZD9668, was also able to reduce IL-1β secretion (**Supplementary Figure S9**)

Altogether, our results indicated that NSPs but not caspase-1 mainly contribute to pro-IL-1β processing in human neutrophils and caspase-1 is necessary for IL-1β secretion, even though the role of this enzyme remains to be determined and deserves future studies.

## Discussion

Previous studies indicated that human neutrophils produce IL-1β and secrete this cytokine by an autophagy-dependent mechanism (10, 18, 19). Our previous data also showed that not only caspase-1 but also NSPs inhibition (NE and/or PR3) reduces IL-1β secretion, however, the role of these enzymes in the mechanisms that lead to IL-1β secretion had not been elucidated (18, 19). Recent studies also indicated that in NLRP3-activated human neutrophils, N-GSDMD oligomerizes on azurophil granule membranes causing leakage of NE into the cytosol that results in secondary cleavage of GSDMD to an alternatively cleaved N-GSDMD product (10). Other studies also showed that NSPs, especially NE, process GSDMD generating fragments able to originate pores that produce HEK cell lysis (24, 25). Additional work also showed that *in vitro* both elastase, CG and PR3 efficiently process pro-IL-1β (15, 26). Thus, considering the abundant content of serine proteases in neutrophils (6), here we investigated the role of caspase-1 and NSPs in the mechanisms involved in pro-IL-1β processing in human neutrophils and those that lead to IL-1β secretion. Our main findings indicated that even though caspase-1 is activated in NLRP3-stimulated human neutrophils and is required for IL-1β secretion, it undergoes rapid inactivation, and instead, NSPs accomplish a major role in pro-IL-1β processing.

We found that AEBSF, a pan-serine protease inhibitor, markedly inhibited IL-1β secretion. Even though we determined this inhibitor blocked autophagy induction, its ability to prevent IL-1β secretion was not entirely associated with this effect but also to its capacity to impair pro-IL-1β processing. In accordance with a previous work (10), which by subcellular fractionation showed the presence of NSPs in the cytosolic fractions of NLRP3-activated murine neutrophils, we detected the presence of active serine proteases in the cytosol of human neutrophils by confocal microscopy. We also determined that these proteases modulated actin dynamic, indicating they are competent to mediate proteolytic actions in the cytosol, such as the pro-IL-1β processing. The major role of NSPs in pro-IL-1β processing in human neutrophils is probably related to the short stability of active caspase-1 in these cells. In fact, our findings indicated that the duration of caspase-1 activity in neutrophils is determined by an intricate weave of regulatory mechanisms that involve its self-inactivation, its inactivation by NSPs and the inflammasome degradation by autophagy.

Previous studies by Schroder’s et al. showed that the inflammasome–caspase-1 complex functions as a holoenzyme that directs the location of caspase-1 activity (14). The authors reported that active caspase-1 species (p46 dimers and p33/10 dimers) can undergo inactivation by a self-cleavage that releases p20/p10 dimers from the inflammasome and ceases the protease activity. Based on the fact they found that murine neutrophils assemble smaller ASC speck than macrophages between 1 and 5 h post-Nigericin stimulation, and they were able to find some active caspase-1 until 8 h post-Nigericin stimulation, the authors concluded that neutrophil caspase-1 activity duration is extended relative to macrophages. However, in human neutrophils we found that a great proportion of caspase-1 undergoes rapid inactivation even earlier than one hour after inflammasome activation. Furthermore, our results suggest that caspase-1 self-inactivation is only one of the mechanisms that control caspase-1 deactivation. In fact, as previously reported in macrophages (21, 27), our findings indicated that inflammasomes can be found colocalizing with autophagosomes, and autophagy also controls the duration of caspase-1 activity. Cytosolic serine proteases also contribute to caspase-1 inactivation. Indeed, when employing AEBSF, which inhibits both autophagy induction and NSPs activity, the addition of FLICA before or after inflammasome activation by ATP did not affect the percentage of cells with active caspase-1 (**Figure 4H**). By contrast, when only autophagy was inhibited by 3-MA or Baf A1, but NSP were still active, a reduction in the percentage of cells with active caspase-1 was observed if FLICA was added 10 min after inflammasome activation, indicating that in this frame of time some caspase-1 underwent cytosolic inactivation (**Figure 4G**). Thus, once in the cytosol, NSPs not only contribute to pro-IL-1β processing but also to caspase-1 inactivation.

Considering previous studies indicating that caspase-1 process GSDMD allowing N-GSDMD to polymerize on azurophil granule membranes leading to NSPs leakage to the cytosol (10), the consequences of this outflow will probably depend on the strength or nature of the stimulus that triggers inflammasome activation. Our findings with neutrophils from patients with a GOF *NLRP3-*mutation suggest that a greater caspase-1 activation might be detrimental for neutrophil IL-1β secretion. This possibility was further supported by results indicating that a stronger inflammasome stimulation with increasing ATP concentrations reduced IL-1β secretion. Considering the presence of many NSPs target sites in IL-1β sequence, it is possible to speculate that those stimuli which induce weak caspase-1 activation will probably allow NSPs to process pro-IL-1β; while those which induce strong activation of caspase-1 might induce a larger NSPs leakage contributing to rapid caspase-1 inactivation and IL-1β degradation. Thus, human neutrophils might modulate its IL-1β-dependent pro-inflammatory potential by means of an intrinsic mechanism that, by limiting the stability of active caspase-1, controls the cytosolic NSPs availability determining whether pro-IL-1β is processed to its active state or degraded.

Our studies showed that inhibition of autophagy also contributes to an increase in caspase-1 activation. This effect appears to be caused by both an augmented activation of the enzyme and a limited remotion of inflammasomes. Previous studies in autophagy-deficient macrophages showed that these cells accumulate dysfunctional mitochondria which release ROS and mitochondrial DNA in the cytosol, leading to NLRP3 activation, an effect that was potentiated by LPS and/or ATP (22, 28). However, mitochondria are scant in human neutrophils and differ from those in other myeloid cells, and the number of copies of mitochondrial DNA is 10-15-fold lower than in peripheral blood mononuclear cells (29). Thus, further studies are required to elucidate if the same events take place in human neutrophils and lead to caspase-1 activation upon autophagy inhibition.

Regarding the role of autophagy as a suppressor of inflammasome activity, our findings are in line with previous work in THP-1 cells, macrophages and monocytes that showed that the activation of inflammasomes lead to autophagy induction and to polyubiquitination of ASC in the inflammasomes, which recruits the autophagic adaptor SQSTM1/p62 resulting in inflammasome degradation by selective autophagy (21). However, we speculate that in macrophages and neutrophils, inflammasome removal by autophagy might have different consequences. In macrophages it might restrain IL-1β secretion, but in neutrophils, it might limit the massive leakage of NSPs from azurophil granules avoiding IL-1β degradation.

Our results showing that autophagy inhibition increased caspase-1 activation even in unstimulated neutrophils, are also in accordance with previous work that suggested that lower autophagy levels might keep inadvertent inflammasome activity in check (21).

Early studies in mice with serine protease activity deficiency, showed a diminished recruitment of neutrophils to air pouches in response to zymosan, which were accompanied by reduced IL-1β levels (30). Additional literature reported a role of NSPs in IL-1β-mediated pathologies in mice models (17). In fact, studies in a mouse model of *P. aeruginosa* corneal infection showed that neutrophils are the primary source of mature IL-1β, and the processing of cytokine’s precursor is dependent on serine proteases, although in this case, neither NLRC4 nor caspase-1 were involved (31). Furthermore, *in vivo* studies also showed that PR3 and elastase can compensate caspase-1 absence for pro-IL-1β processing both in a serum transfer–induced arthritis model and in the monosodium urate monohydrate crystal–induced peritonitis model (32). Results of our study add to previous literature elucidating one mechanism by which NSPs might contribute to different IL-1β-mediated diseases dominated by neutrophil recruitment.

Altogether, the results of our study indicate that NSPs regulate the unconventional IL-1β secretion in human neutrophils. Furthermore, caspase-1 activation is necessary but not sufficient for IL-1β secretion as it appears to play a minor role in pro-IL-1β processing probably because it is rapidly inactivated after inflammasome activation. Autophagy not only controls IL-1β secretion but also the frame time caspase-1 is active by targeting inflammasomes for degradation.

Our findings reveal the usefulness to study potential therapeutic interventions directed to inactivate NSPs, which might be more cost-effective than targeting IL-1β to control inflammation in those pathologies where neutrophil-derived IL-1β plays a key pathogenic role, without extensively compromising broad host defense against pathogens.

## Data availability statement

The original contributions presented in the study are included in the article/**Supplementary Material**. Further inquiries can be directed to the corresponding author.

## Ethics statement

The studies involving human participants were reviewed and approved by Ethical Committee of the “Institutos de la Academia Nacional de Medicina”. Written informed consent to participate in this study was provided by the participants or the participants' legal guardian/next of kin.

## Author contributions

IK, FS and CS conducted most of the assays; IK, FS and AT designed the study, analyzed the data, and wrote the paper; NZ, CJ, DR, DVA and JG performed some experiments; SD, JY, MM and GB, conducted some assays with patients and/or analyzed data; FF programmed the image quantification macros, and performed microscopy acquisitions together with IK and AT; VG contributed to patients’ sequences analysis; MK was involved in clinical workup of patients; and MG provided scientific expertise and analyzed data. All authors contributed to the article and approved the submitted version.

## Funding

This work was supported by grants from Agencia Nacional de Promoción Científica y Tecnológica, Ministerio de Ciencia, Tecnología e Innovación, Argentina [PICT2016-1418 and PICT-2018-02338 (A T); and PICT 2018-0546 (MG)], and Consejo Nacional de Investigaciones Científicas y Técnicas (CONICET), Buenos Aires, Argentina. This study also received funding from Novartis (MM). The funder was not involved in the study design, collection, analysis, interpretation of data, the writing of this article or the decision to submit it for publication.

## Conflict of interest

MM receives a scientific research grant from Novartis; MK gives lectures for Pfizer and Novartis.

The remaining authors declare that the research was conducted in the absence of any commercial or financial relationships that could be construed as a potential conflict of interest.

## Publisher’s note

All claims expressed in this article are solely those of the authors and do not necessarily represent those of their affiliated organizations, or those of the publisher, the editors and the reviewers. Any product that may be evaluated in this article, or claim that may be made by its manufacturer, is not guaranteed or endorsed by the publisher.

## References

[1] Ley K Hoffman HM Kubes P Cassatella MA Zychlinsky A Hedrick CC. Neutrophils: New insights and open questions. Sci Immunol (2018) 3(30):eaat4579. doi: 10.1126/sciimmunol.aat4579 30530726

[2] Tamassia N Bianchetto-Aguilera F Arruda-Silva F Gardiman E Gasperini S Calzetti F. Cytokine production by human neutrophils: Revisiting the “dark side of the moon.” Eur J Clin Invest (2018) 48(Supp.2):e12952. doi: 10.1111/eci.12952 29772063

[3] Chan AH Schroder K. Inflammasome signaling and regulation of interleukin-1 family cytokines. J Exp Med (2020) 217(1):1–10. doi: 10.1084/jem.20190314 PMC7037238 31611248

[4] Rørvig S Østergaard O Heegaard NHH Borregaard N. Proteome profiling of human neutrophil granule subsets, secretory vesicles, and cell membrane: correlation with transcriptome profiling of neutrophil precursors. J Leukoc Biol (2013) 94(4):711–21. doi: 10.1189/jlb.1212619 23650620

[5] Majewski P Majchrzak-Gorecka M Grygier B Skrzeczynska-Moncznik J Osiecka O Cichy J. Inhibitors of serine proteases in regulating the production and function of neutrophil extracellular traps. Front Immunol (2016) 7:1–10. doi: 10.3389/fimmu.2016.00261 PMC4928128 27446090

[6] Kettritz R. Neutral serine proteases of neutrophils. Immunol Rev (2016) 273(1):232–48. doi: 10.1111/imr.12441 27558338

[7] Liu P Ye K Kasorn A Cao S Zhou J Gong H. Proteinase 3–dependent caspase-3 cleavage modulates neutrophil death and inflammation. J Clin Invest (2014) 124(10):4445–58. doi: 10.1172/JCI76246 PMC4191030 25180606

[8] Papayannopoulos V Metzler KD Hakkim A Zychlinsky A. Neutrophil elastase and myeloperoxidase regulate the formation of neutrophil extracellular traps. J Cell Biol (2010) 191(3):677–91. doi: 10.1083/jcb.201006052 PMC3003309 20974816

[9] Metzler KD Goosmann C Lubojemska A Zychlinsky A. A myeloperoxidase-containing complex regulates neutrophil elastase release and actin dynamics during NETosis. Cell Rep (2014) 8(3):883–96. doi: 10.1016/j.celrep.2014.06.044 PMC4471680 25066128

[10] Karmakar M Minns M Greenberg EN Diaz-Aponte J Pestonjamasp K Johnson JL. N-GSDMD trafficking to neutrophil organelles facilitates IL-1β release independently of plasma membrane pores and pyroptosis. Nat Commun (2020) 11(1):1–14. doi: 10.1038/s41467-020-16043-9 32371889 PMC7200749

[11] Martinon F Burns K Rg Tschopp J. The inflammasome: A molecular platform triggering activation of inflammatory caspases and processing of proIL-that they possess several distinct protein/protein inter-action domains which are used to assemble large multi-component complexes. apaf-1, for e. Mol Cell (2002) 10(2):417–26. doi: 10.1016/s1097-2765(02)00599-3 12191486

[12] Ketelut-Carneiro N Fitzgerald KA. Inflammasomes. Curr Biol (2020) 30(12):R689–94. doi: 10.1016/j.cub.2020.04.065 32574626

[13] He Y Hara H Núñez G. Mechanism and regulation of NLRP3 inflammasome activation. Trends Biochem Sci (2016) 41(12):1012–21. doi: 10.1016/j.tibs.2016.09.002 PMC5123939 27669650

[14] Boucher D Monteleone M Coll RC Chen KW Ross CM Teo JL. Caspase-1 self-cleavage is an intrinsic mechanism to terminate inflammasome activity. J Exp Med (2018) 215(3):827–40. doi: 10.1084/jem.20172222 PMC5839769 29432122

[15] Greten FR Arkan MC Bollrath J Hsu LC Goode J Miething C. NF-κB is a negative regulator of IL-1β secretion as revealed by genetic and pharmacological inhibition of IKKβ. Cell. (2007) 130(5):918–31. doi: 10.1016/j.cell.2007.07.009 PMC2134986 17803913

[16] Coeshott C Ohnemus C Pilyavskaya A Ross S Wieczorek M Kroona H. Converting enzyme-independent release of tumor necrosis factor α and IL-1β from a stimulated human monocytic cell line in the presence of activated neutrophils or purified proteinase 3. Proc Natl Acad Sci U S A. (1999) 96(11):6261–6. doi: 10.1073/pnas.96.11.6261 PMC26869 10339575

[17] Netea MG van de Veerdonk FL van der Meer JWM Dinarello CA Joosten LAB. Inflammasome-independent regulation of IL-1-Family cytokines. Annu Rev Immunol (2015) 33(1):49–77. doi: 10.1146/annurev-immunol-032414-112306 25493334

[18] Gabelloni ML Sabbione F Jancic C Fuxman Bass J Keitelman I Iula L. NADPH oxidase derived reactive oxygen species are involved in human neutrophil IL-1β secretion but not in inflammasome activation. Eur J Immunol (2013) 43(12):3324–35. doi: 10.1002/eji.201243089 23963575

[19] Iula L Keitelman IA Sabbione F Fuentes F Guzman M Galletti JG. Autophagy mediates interleukin-1β secretion in human neutrophils. Front Immunol (2018) 9:1–14. doi: 10.3389/fimmu.2018.00269 PMC5825906 29515581

[20] Van Gijn ME Ceccherini I Shinar Y Carbo EC Slofstra M Arostegui JI. New workflow for classification of genetic variants’ pathogenicity applied to hereditary recurrent fevers by the international study group for systemic autoinflammatory diseases (INSAID). J Med Genet (2018) 55(8):530–7. doi: 10.1136/jmedgenet-2017-105216 29599418

[21] Shi CS Shenderov K Huang NN Kabat J Abu-Asab M Fitzgerald K a. Activation of autophagy by inflammatory signals limits IL-1β production by targeting ubiquitinated inflammasomes for destruction. Nat Immunol (2012) 13(3):255–63. doi: 10.1038/ni.2215 PMC4116819 22286270

[22] Nakahira K Haspel JA Rathinam V Lee S-J Dolinay T Lam HC. Autophagy proteins regulate innate immune responses by inhibiting the release of mitochondrial DNA mediated by the NALP3 inflammasome. Nat Immunol (2011) 12(3):222–30. doi: 10.1038/ni.1980 PMC3079381 21151103

[23] Afonina IS Müller C Martin SJ Beyaert R. Proteolytic processing of interleukin-1 family cytokines: Variations on a common theme. Immunity. (2015) 42(6):991–1004. doi: 10.1016/j.immuni.2015.06.003 26084020

[24] Kambara H Liu F Zhang X Liu P Bajrami B Teng Y. Gasdermin d exerts anti-inflammatory effects by promoting neutrophil death. Cell Rep (2018) 22(11):2924–36. doi: 10.1016/j.celrep.2018.02.067 PMC5878047 29539421

[25] Sollberger G Choidas A Burn GL Habenberger P Di Lucrezia R Kordes S. Gasdermin d plays a vital role in the generation of neutrophil extracellular traps. Sci Immunol (2018) 3(26):eaar6689. doi: 10.1126/sciimmunol.aar6689 30143555

[26] Hazuda DJ Strickler J Kueppers F Simon PL Young PR. Processing of precursor interleukin 1 beta and inflammatory disease. J Biol Chem (1990) 265(11):6318–22. doi: 10.1016/S0021-9258(19)39328-7 2156847

[27] Harris J Lang T Thomas JPW Sukkar MB Nabar NR Kehrl JH. Autophagy and inflammasomes. Mol Immunol (2017) 86:10–5. doi: 10.1016/j.molimm.2017.02.013 28249679

[28] Zhou R Yazdi AS Menu P Tschopp J. A role for mitochondria in NLRP3 inflammasome activation. Nature (2011) 469(7329):221–6. doi: 10.1038/nature09663 21124315

[29] Maianski N a Geissler J Srinivasula SM Alnemri ES Roos D Kuijpers TW. Functional characterization of mitochondria in neutrophils: a role restricted to apoptosis. Cell Death Differ (2004) 11(2):143–53. doi: 10.1038/sj.cdd.4401320 14576767

[30] Adkison AM Raptis SZ Kelley DG Pham CTN. Dipeptidyl peptidase I activates neutrophil-derived serine proteases and regulates the development of acute experimental arthritis. J Clin Invest (2002) 109(3):363–71. doi: 10.1172/JCI0213462 PMC150852 11827996

[31] Karmakar M Sun Y Hise AG Rietsch A Pearlman E. IL-1β processing during pseudomonas aeruginosa infection is mediated by neutrophil serine proteases and is independent of NLRC4 and caspase-1. J Immunol (2013) 189(9):4231–5. doi: 10.4049/jimmunol.1201447 PMC3482477 23024281

[32] Guma M Ronacher L Liu-Bryan R Takai S Karin M Corr M. Caspase 1-independent activation of interleukin-1beta in neutrophil-predominant inflammation. Arthritis Rheum (2009) 60(12):3642–50. doi: 10.1002/art.24959 PMC2847793 19950258

