# Supplementary material for: The interplay between serine proteases and caspase-1 regulates the autophagy-mediated secretion of Interleukin-1 beta in human neutrophils
Source: Front Immunol. 2022 Aug 25;13:832306. doi: 10.3389/fimmu.2022.832306 (PMC9458071; doi:10.3389/fimmu.2022.832306)

Supplementary material

Legend to supplementary movie 1. Neutrophils ($4x10^5$) were seeded on 0,01% Poly-L-lysin-treated Cellview glass bottom dish, stimulated with LPS from *Escherichia coli* O111:B4 (150 ng/mL) in a humidified atmosphere containing 5% CO_2 for 2 h. Then, inflammasome activation was triggered by ATP (2.5 mM). Ten min later FLICA was added, and cells were monitored for 1 h by live cell imaging. Images corresponding to FLICA channel (green) and differential interface contrast (DIC) were acquired every 5 sec.

Legend to supplementary movie 2. Neutrophils ($4x10^5$) were seeded on 0,01% Poly-L-lysin-treated Cellview glass bottom dish, stimulated with LPS from *Escherichia coli* O111:B4 (1 µg/ml), stained with the fluorescent probes FAM-FLISP and Lysotracker red and treated with ATP (2.5 mM; t= 2h). The cells, incubated under an atmosphere containing 5% CO_2 at 37°C, were monitored by live cell imaging in three different channels: differential interface contrast (DIC), the cell permeable dye FAM-FLISP-FLCK (green) and the Lysotracker red channel (red). Images were acquired every 123 sec. The green fluorescence inside the cells represents active NSPs. Fluorescence outside the cells is emitted by the probe that had to be present along the imaging to increase the sensitivity of the assay. White arrows show active NSPs with a diffuse distribution; gray arrows show active NSPs with a vesicular distribution.

Legend to supplementary movie 3. Neutrophils ($5x10^5$) were stained with CellBrite fix 640 and seeded on 0,01% Poly-L-lysin-treated Cellview glass bottom dish. Then were stained with Lysotracker red and FAM-FLISP and stimulated with LPS from *Escherichia coli* O111:B4 (150 ng/ml). At t=2 h, cells were treated with ATP (2.5 mM) and imaged inside a camera under an atmosphere containing 5% CO_2 at 37°C in three different channels: differential interface contrast (DIC), the cell permeable dye FAM-FLISP-FLCK (green) and the Lysotracker red channel (red). Images were acquired every 63.3 sec. Only FAM-FLISP and Lysotracker red fluorescence are shown. The green fluorescence inside the cells represents active NSPs. White arrows show active NSPs with a diffuse distribution; the yellow arrow show active NSPs within a Lysotracker red containing vesicle.

Supplementary Figure S1. Representative dot plots of viability assays of neutrophils left unstimulated, or stimulated with LPS+ATP, in the presence or absence of AEBSF 0.35 mM or 1 mM. Cell viability was evaluated by Annexin V/Propidium iodide staining and flow cytometry at 5 h post LPS stimulation.

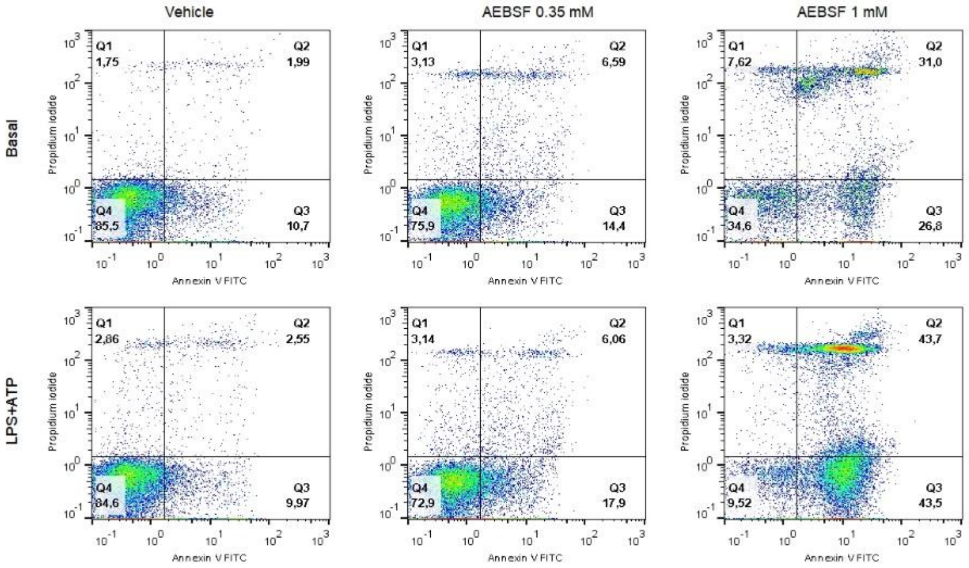

Supplementary Figure S2. Neutrophils were stimulated or not with LPS and 2 h later were treated with ATP. Ten minutes later they were incubated with the FAM-FLICA probe and fluorescence was determined at 2 h 40 min by flow cytometry. Graph depicts the median fluorescence intensity value of different donors. Values corresponding to each single donor are shown joined with a line.

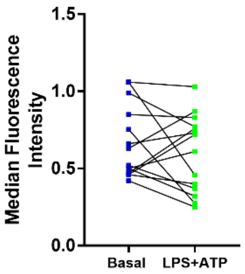

Supplementary Figure S3. Neutrophils from patients and aged-matched control donors were stimulated or not with LPS (t= 0) and 2 h later were stimulated or not with ATP. At 5 h post-LPS stimulation IL-1β concentrations in culture supernatants were determined by ELISA. Each bar represents the mean+SEM of the cytokine concentrations of an individual donor evaluated in triplicate.

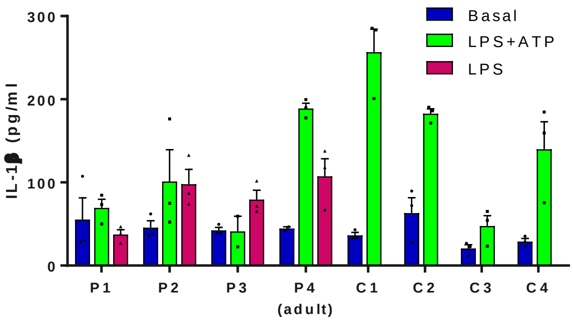

Supplementary Figure S4. Peripheral blood mononuclear cells (PBMCs) from control healthy donors (C) or from patients with the *NLRP3*-gain-of-function (GOF) mutation c.1322C>T (p.Ala441Val) were isolated after gradient centrifugation over Ficoll-Paque (Sigma), washed thrice and suspended in RPMI 1640 supplemented with 10 mM of L-glutamine, 10 mg/ml of streptomycin, and 10% of Fetal Bovine Serum (Gibco, USA). Cells (10^6/ml) were stimulated with MDP (200 ng/ml; InvivoGen,USA) plus MSU crystals (250 μg/ml) prepared as previously described (DOI: 10.1159/000460293) for 3 h at 37º C in 5% CO_2. Afterwards, supernatants were collected and IL-1β concentrations were determined by ELISA.

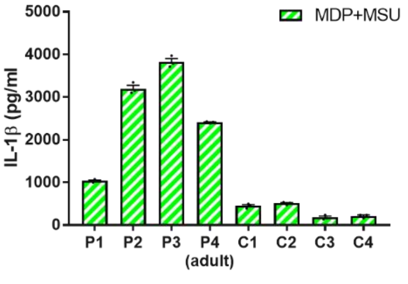

Supplementary Figure S5. Neutrophils were pre-treated with Ac-YVAD-CMK (50μM; A) or VX-765 (50 μM; B), stimulated or not with LPS and 2 h later were treated or not with ATP. At 5 h post-LPS stimulation supernatants were collected and IL-1β concentrations were determined by ELISA. *p<0.05 One way ANOVA.

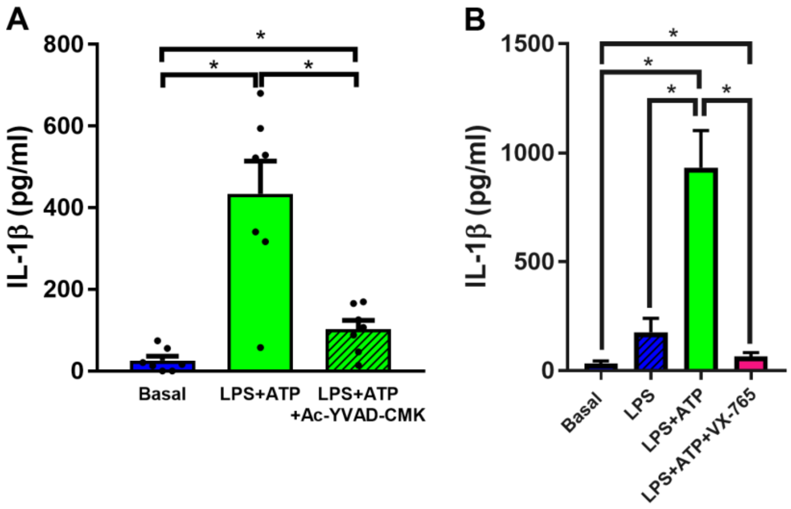

Supplementary Figure S6. Predicted cleavage sites in the human pro-IL-1β sequence according to Expasy PeptideCutter for elastase. For comparison caspase-1 cleavage sites are also depicted.

Expasy

PeptideCutter

The sequence to investigate:

```
        10         20         30         40         50         60
MAEVPELASE MMAYYSGNED DLFFEADGPK QMKCSFQDLD LCPLDGGIQL RISDHHYSKG

        70         80         90        100        110        120
FRQAASVVVA MDKLRKMLVP CPQTFQENDL STFFPFIFEE EPIFFDTWDN EAYVHDAPVR

       130        140        150        160        170        180
SLNCTLRDSQ QKSLVMSGPY ELKALHLQGQ DMEQQVVFSM SFVQGEESND KIPVALGLKE

       190        200        210        220        230        240
KNLYLSCVLK DDKPTLQLES VDPKNYPKKK MEKRFVFNKI EINNKLEFES AQFPNWYIST

       250        260
SQAENMPVFL GGTKGGQDIT DFTMQPVSS
```

The sequence is 269 amino acids long.

Available enzymes

The enzyme(s) that you have chosen:

- Caspase1
- Neutrophil elastase

You have chosen to display all possible cleaving enzymes.

These enzymes cleave the sequence:

Name of enzyme	No. of cleavages	Positions of cleavage sites
Caspase1	2	27 116
Neutrophil elastase	30	2 4 8 13 26 64 65 67 68 69 70 79 112 114 117 119 135 144 156 157 163 174 175 188 201 216 231 243 248 267

These are the cleavage sites of the chosen enzymes and chemicals mapped onto the entered protein sequence:

- You have chosen a block size of **60** for the map.
- Please note that the cleavage occurs at the **right side** (C-terminal direction) of the marked amino acid.
- You have the possibility to display the results of a single enzyme by **mouseclicking** on the respective enzyme name in the map.

```
             Elast
        Elast    |
    Elast   |    |         Casp1
  Elast |   |    |        Elast|

      | |   |    |            ||
     MAEVPELASEMMAYYSGNEDDLFFEADGPKQMKCSFQDLDLCPLDGGIQLRISDHHYSKG
  1  ---------+---------+---------+---------+---------+---------+   60
```

```
          Elast
         Elast|                                            Elast
        Elast||                                          Elast |
       Elast|||                                         Casp1| |
     Elast ||||                                       Elast ||  |
    Elast| ||||    Elast                            Elast | || |

        || ||||        |                                |  | || |
     FRQAASVVVAMDKLRKMLVPCPQTFQENDLSTFFPFIFEEEPIFFDTWDNEAYVHDAPVR
 61  ---------+---------+---------+---------+---------+---------+   120
```

```
                                     Elast             Elast
               Elast    Elast       Elast| Elast      Elast|

                   |        |           ||     |          ||
     SLNCTLRDSQQKSLVMSGPYELKALHLQGQDMEQQVVFSMSFVQGEESNDKIPVALGLKE
121  ---------+---------+---------+---------+---------+---------+   180
```

```
        Elast        Elast          Elast          Elast

            |            |              |              |
     KNLYLSCVLKDDKPTLQLESVDPKNYPKKKMEKRFVFNKIEINNKLEFESAQFPNWYIST
181  ---------+---------+---------+---------+---------+---------+   240
```

```
        Elast
   Elast    |              Elast

       |    |                  |
     SQAENMPVFLGGTKGGQDITDFTMQPVSS
241  ---------+---------+---------   269
```

Supplementary Figure S7. (A and B) Neutrophils were pre-treated for 30 min in the presence or absence of AEBSF (1 mM), Ac-YVAD-CMK (50 µM), or their combination, then cultured with or without LPS for 2 h and after were stimulated or not with ATP (2.5 mM) for another 1.5 h. Then, whole cell extracts were subjected to Western blot (A) and supernatants collected for ELISA quantification (B). (A) Representative Western blot assay (n=4) of pro-IL-1β and mature IL-1β expression and (B) IL-1β concentration in culture supernatants of the same cells from which lysates were immunoblotted. Note: The image corresponding to the membrane was cut and spliced between the basal and LPS+ATP lane.

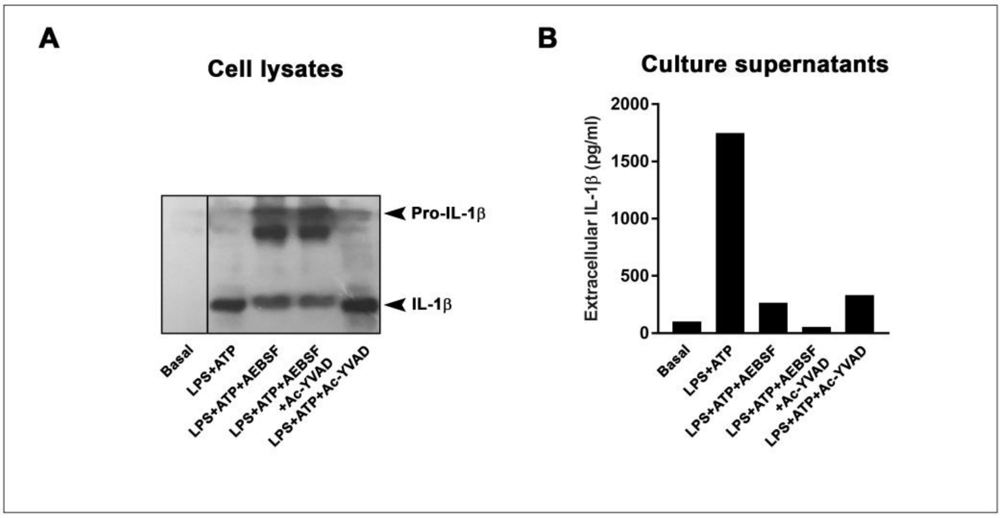

Supplementary Figure S8. Intracellular pro-IL-1β expression in neutrophils pretreated or not with AEBSF (0.35 mM), 30 min later stimulated with LPS and 2 h after with ATP and evaluated at 3.5 h by immunostaining with an anti-pro-IL-1β specific antibody and flow cytometry. Histograms are representative of experiments with 2 donors.

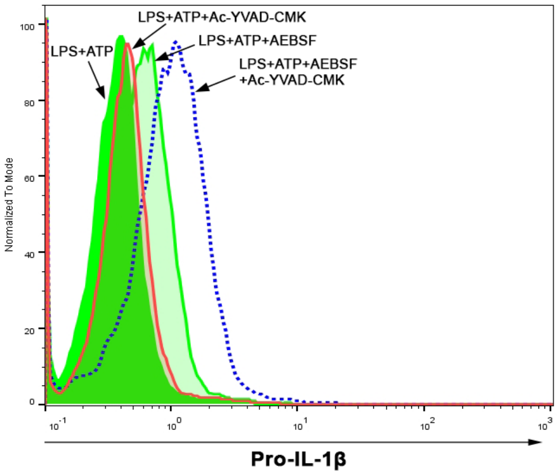

Supplementary Figure S9. Neutrophils were pre-treated with AZD9668 (100 µM) or vehicle, stimulated with LPS (150 ng/ml) and 2 h later were treated with ATP (2.5 mM). At 5 h post-LPS stimulation supernatants were collected and IL-1β concentrations were determined by ELISA. *p<0.05; Student t test.

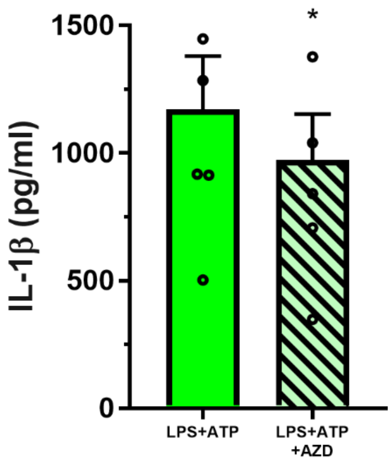

Supplementary Figure S10. Raw western blots corresponding to Figures 2, 4 and 8.

LC3B Santa Cruz #28266

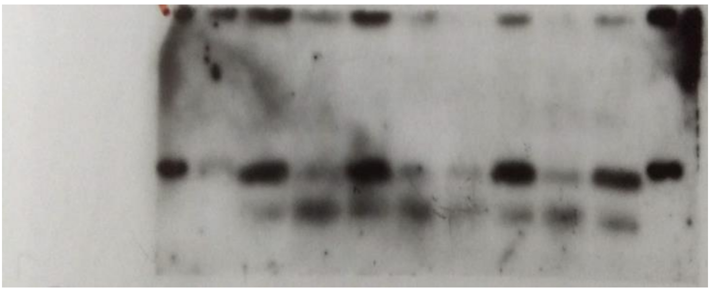

MPO Dako cat.#A0398

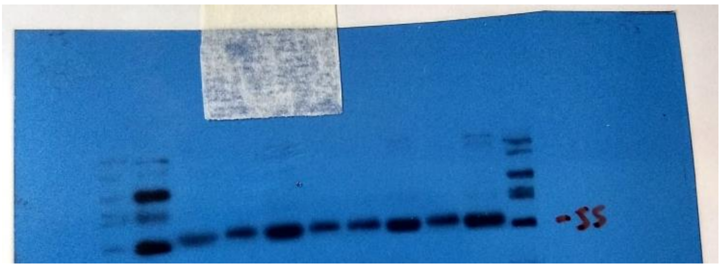

Caspase-1 Ab Santa Cruz #515

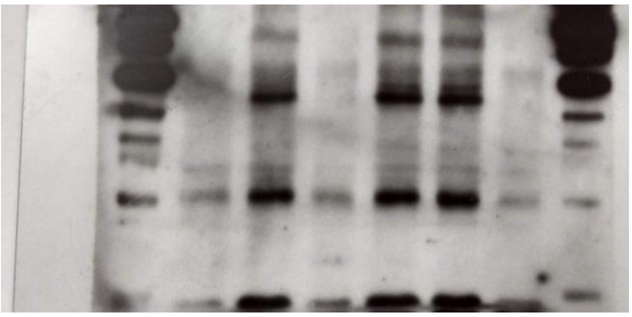

MPO Dako cat.#A0398

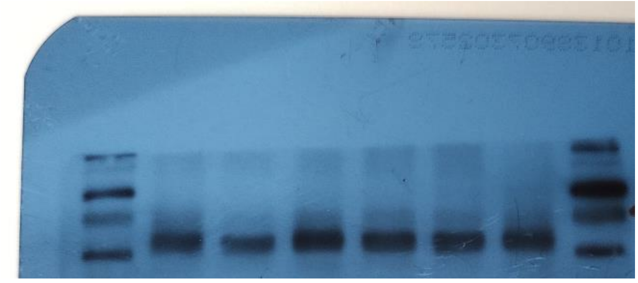

Caspase-1 Ab Santa Cruz #56036

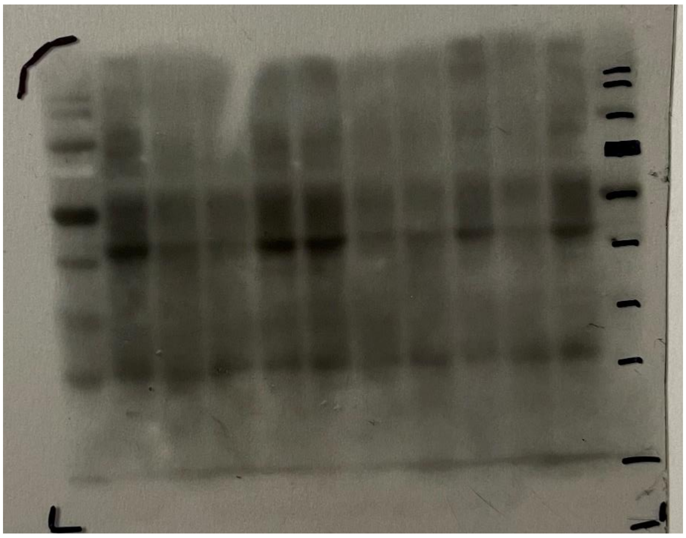

MPO Dako cat.#A0398

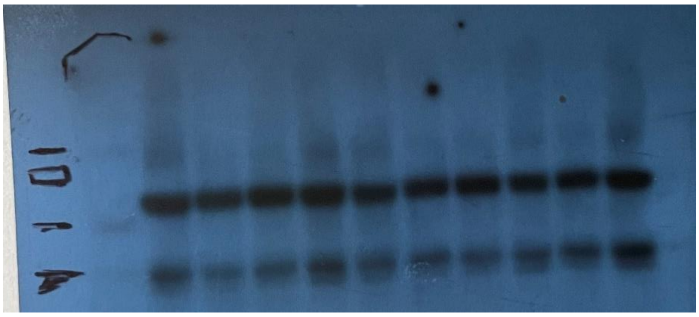

IL-1β Ab Novus Biologicals #NB600-633

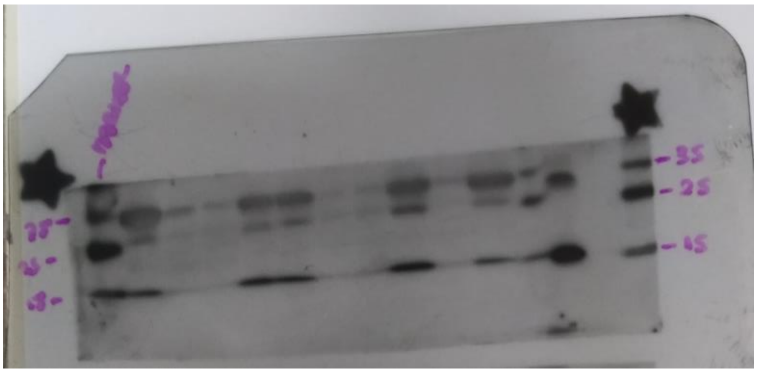

MPO Dako cat.#A0398

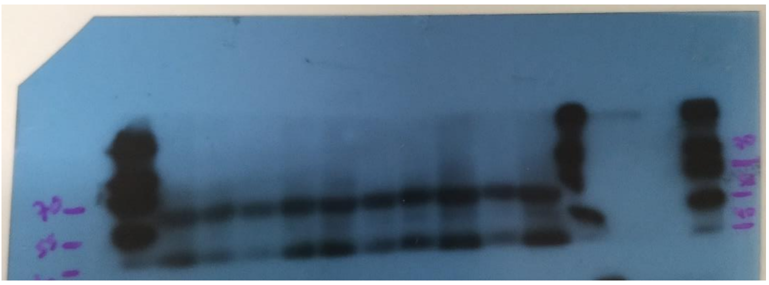

IL-1β Ab Santa Cruz #H153

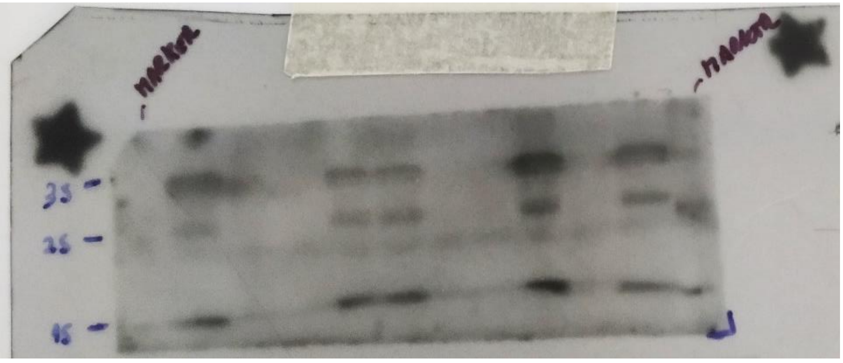

MPO Dako cat.#A0398

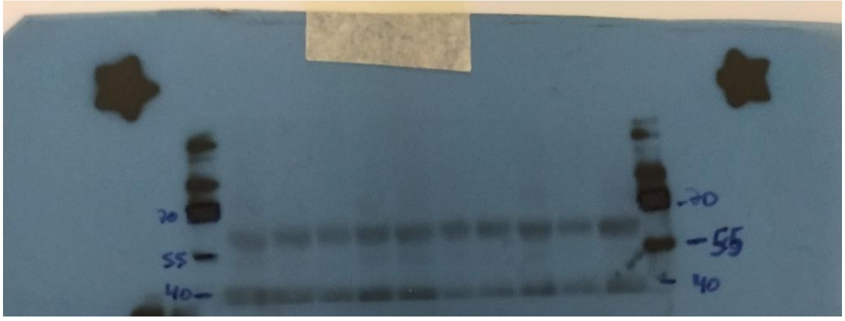

Supplement: Supplementary file 1 [file DataSheet_1.pdf]
